# Cell-specific Cahn-Hilliard models predict condensed fates of the chromosomal passenger complex

**DOI:** 10.1371/journal.pcbi.1014568

**Published:** 2026-08-03

**Authors:** Sarah M. Groves, Min-Jhe Lu, Astrid Catalina Alvarez-Yela, Monserrat Gerardo-Ramírez, P. Todd Stukenberg, John S. Lowengrub, Kevin A. Janes

**Affiliations:** 1 Department of Biomedical Engineering, University of Virginia, Charlottesville, Virginia, United States of America; 2 Institute of Computational and Modeling Science, National Tsing Hua University, Hsinchu, Taiwan; 3 Department of Mathematics, University of California at Irvine, Irvine, California, United States of America; 4 Department of Biochemistry and Molecular Genetics, University of Virginia, Charlottesville, Virginia, United States of America; 5 Department of Biomedical Engineering, University of California at Irvine, Irvine, California, United States of America; Indian Institute of Technology Hyderabad, INDIA

## Abstract

Biomolecular condensates create dynamic subcellular compartments that alter systems-level properties of the networks surrounding them. Standard reaction-diffusion models of systems biology cannot define where these compartments emerge nor track how they evolve. One alternative physicochemical model of soluble and condensed states in space and time is the Cahn-Hilliard equation, which specifies a diffuse interface between the two phases. Customized numerical approaches required to solve this equation are absent from computing environments often used for systems biology, however, and the equation’s interfacial energy coefficient lacks empirical constraints. Here, using two complementary numerical strategies, we built stable, self-consistent Cahn-Hilliard solvers in three common systems-biology programming languages. The algorithms simulated the complete time evolution of condensed droplets as they dissolved or persisted, relating critical equilibrium droplet size to the Cahn-Hilliard interfacial energy coefficient. We applied this universal relationship to the chromosomal passenger complex, a multi-protein assembly that reportedly condenses on mitotic chromosomes. The fully constrained Cahn-Hilliard simulations predicted spatiotemporal dewetting and coarsening behaviors that matched experiments in cell types with different interfacial energy coefficients. Together, these results suggest how initially variegated recruitment yields robust localization of the chromosomal passenger complex to the inner centromere by the end of prometaphase. More generally, the Cahn-Hilliard equation tests whether condensate dynamics behave as a simple phase-separated liquid, and its numerical solutions advance generalized modeling of biomolecular systems.

## Author summary

Some biomolecules separate into condensed and soluble phases within cells; it is important to study the behavior of these compartments with mathematical models. From chemical physics, there is a macroscale theory for phase-separated systems, but the governing equation is difficult to solve numerically with standard approaches. Here, we encoded and verified a pair of specialized solvers in three programming languages widely used by systems biologists. The math behind each solver is different, enabling them to crosscheck one another for self-consistency. We used the solvers to define a previously unknown relationship within the governing equation that holds for any phase-separated system. When applied to a protein complex whose condensation is debated, the numerical solutions yielded absolute predictions that were remarkably consistent with experiments. This work provides a combined computational–experimental path to parameterizing the governing equation for generalized models of biomolecular condensates.

## Introduction

Phase separation arises whenever random-walk diffusion is countered by attractive forces that are strong enough to offset the energetic penalty of forming an interface between phases. In biology, such attractive forces may be biomolecular [1,2], adhesive [3,4], mechanical [4–6], or unknown [7]. Phase-separating behavior has been documented at biological length scales ranging from subdomains of organellar membranes [8] to entire ecological communities [9]. One area of interest for systems biology is the condensation of biomolecules as 0.1–1 µm droplets within cells, which are thought to change the emergent properties of networks by insulation [10], kinetic modulation [11], and buffering [12].

Condensed two-phase systems can be modeled as a free-boundary problem that posits a zero-thickness interface with a surface tension and accompanying force balance. However, this approach is problematic at small length scales, which must be considered when very small droplets form, as in the Rayleigh instability [13,14], or dissolve, as in Ostwald ripening [15]. An alternative that dates back to Poisson is to consider a diffuse boundary, which changes between phases over a length scale that emerges from an imbalance in intermolecular forces [16]. An order parameter identifies one of the chemical states (Box 1), and governing equations follow an energy variational approach. One classic example is the Cahn-Hilliard equation [17], which evolves by diffusion arising from the variational derivative of a free energy. The Cahn-Hilliard free energy contains a local function with two minima (one for each chemical state; Box 1) and a gradient term that penalizes the formation of interfacial regions (Box 2). The Cahn-Hilliard model is appealing for two-phase chemical transport because it conserves mass and dissipates energy toward a thermodynamic minimum. Indeed, many of the biological examples cited above have been modeled as Cahn-Hilliard systems.

For most two-phase phenomena, there is not an exact solution for the Cahn-Hilliard equation. Numerical approximations are possible, but they require specialized approaches to address space/time-step restrictions, local nonlinearities, and nonconvexity. Published algorithms demonstrate proof of concept but historically do not include freely available code for redeployment in computational biology. Rare exceptions [18–20] are limited to specific boundary conditions that may not always be biologically appropriate. A general toolkit for Cahn-Hilliard models may facilitate its application to other examples of biological condensation [7], including settings where phase separation is actively debated.

One such example is the chromosomal passenger complex (CPC), a heterotetramer that coordinates mitotic events by concentrating at the inner centromere during (pro)metaphase [21]. When recruited to chromatin above a critical concentration, the CPC has been suggested to form a biomolecular condensate that is critical for its inner centromeric functions [22]. However, cell biology techniques are limited in their ability to distinguish true condensates from other protein-concentrating interactions, and phase separability of the CPC was recently questioned [23]. One potentially discriminating test is to ask whether the CPC behaves as a Cahn-Hilliard fluid on mitotic chromatin.

Toward modeling the dynamics of biomolecular condensates within cells [24,25], here we assemble and validate a multi-language suite [26] of numerical methods for solving the Cahn-Hilliard equation. The algorithms are stable, self-consistent, and computationally suitable for spatial meshes of up to 2^9^ x 2^9^ = 262,144 elements. Applying the methods to hundreds of long-term, high-resolution Cahn-Hilliard simulations, we define the critical equilibrium radius (*R*_*eq*_) for a stable, condensed droplet across a tenfold range of plausible diffuse interfaces. Focusing on the CPC, we estimate *R*_*eq*_ and with the corresponding diffuse interface simulate different recruitment patterns as a Cahn-Hilliard process. We find that the dewetting and ripening characteristics of the CPC are remarkably consistent with theory without any further parameter fitting or model refinements. This work provides access to reliable software and approaches for abstracting dynamic interfaces in biological systems.

## Results

### Modeling emergent subcellular compartments with the Cahn-Hilliard equation

Systems models of cell biology routinely encode nuclei, mitochondria, endosomes, and other organelles as subcompartments that are static and prespecified by membranes [27–30]. Biomolecular condensates also compartmentalize but with shapes and sizes that are much more dynamic because of interfacial phenomena between condensed and soluble phases [24,31]. Mathematically, interfacial free energy can be formally coupled to the energetics of weak intermolecular interactions [31] (Box 1) and diffusion in space and time through the Cahn-Hilliard equation (Box 2) [17]. Dynamics of the Cahn-Hilliard equation naturally evolve to an equilibrium solution that minimizes the free energy of the overall system. The Cahn-Hilliard equation also conserves mass—solute diffuses freely within the system and can transition between condensed and soluble chemical states (Box 1), but the overall amount remains constant. This is advantageous for modeling biomolecular condensates that evolve on time scales of seconds to minutes (Box 2) [24,31].

### Numerical solvers for the Cahn-Hilliard equation

The energy-minimizing and mass-conserving properties of the Cahn-Hilliard equation set criteria for the accuracy of its numerical solutions. The Cahn-Hilliard equation is intrinsically stiff, because the fast dynamics of interfaces must be considered along with the slower dynamics of phases (Box 2). Additional stiffness challenges arise from the interfacial free energy term embedded within the generalized chemical potential (Eq 7), which results in a fourth-order spatial derivative that is burdensome for standard solvers. Maintaining energy dissipation and mass stability using standard methods requires very-fine spatial meshes, very-small time steps, or global linearizations, any of which adds computational burden.

Various custom methods solve the Cahn-Hilliard equation accurately and efficiently through different numerical schemes (reviewed in [32]), but none are available with biology-focused users in mind. Current solvers are derived mathematically and described in pseudocode or illustrative scripts rather than functions packaged for general use. We thus developed generalized code for one finite-difference method and one spectral method in three widely used programming languages for computational biology: Python [33], MATLAB, and Julia [34]. Our finite-difference method adapts the nonlinear multigrid (NMG; Box 3) approach of Lee et al. [18]. The complementary spectral method uses the scalar auxiliary variable (SAV; Box 3) approach to gradient flows by Shen et al. [35], which can be similarly accurate and efficient. Together, NMG and SAV provide a versatile pair of Cahn-Hilliard solvers for biomolecular condensate systems.

**Fig 1:**
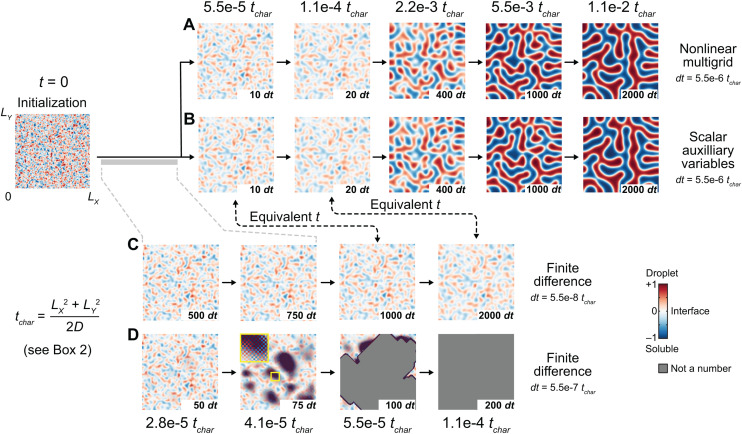
Practical simulations of the Cahn-Hilliard equation require customized solvers. **(A, B)** Time evolution of spinodal decomposition solved by nonlinear multigrid (A) or scalar auxiliary variables (B) over 1.1e-2 characteristic times (*t*_*char*_) defined by the length of *X* and *Y* spatial domains (*L*_*X*_, *L*_*Y*_) and system diffusivity (*D*). Both solvers used a 5.5e-6 time step (*dt*) relative to *t*_*char*_. **(C)** Time evolution for the same spinodal decomposition solved by finite difference using a 100-fold smaller *dt* (5.5e-8 *t*_*char*_) for 1% of the duration in (A) and (B). **(D)** Numerical instabilities triggered by the finite difference solver when using a 10-fold smaller *dt* (5.5e-7 *t*_*char*_) than in (A) and (B). Defects appear as checkerboards in the mesh (inset) and become undefined (gray). Simulations were initialized as a random mixture of ±1 chemical states on a 2^7^ square mesh and smoothed before iterating with Neumann boundary conditions (Materials and Methods). Alternative initializations and boundary conditions are shown in S1 Fig.

Because NMG and SAV are very different algorithmically (Box 3), it was important to confirm that both packages yield the same numerical solutions. As an initial condition, we used a smoothed random mesh of ±1 chemical states (Fig 1, left; Box 1; and Materials and Methods), which spontaneously undergoes a spinodal decomposition into two phases. After ~1% of characteristic time for the system (*t*_*char*_; Eq 11), both NMG and SAV converged to a slowly evolving solution with gradually decreasing energy and no overall change in mass (Figs 1A–B and S1, Panels A–B). The resulting spatial pattern resembles the trajectory of biomolecular condensates triggered to self-assemble on lipid bilayers [36]. We compared results of NMG and SAV to a standard finite-difference implementation of the forward Euler method and successfully captured the first 0.01% of *t*_*char*_ by taking 100-fold smaller time steps (*dt*; Fig 1C). Any larger time steps caused spatial defects in the finite-difference solution along with drastic deviations in energy and mass compared to the true solution (Figs 1D and S1, Panels A–B). Implicit time steps yielded stable solutions at the expense of overall computation speed (S1 Fig, Panels C–D). NMG and SAV were very similar for alternative boundary conditions and smoothing initializations: between solvers, the root mean squared error was less than 0.02 for unsmoothed spinodal decompositions and less than 0.0125 when initial conditions were smoothed (S1 Fig, Panels E–J). These results verified the self-consistency of our NMG–SAV implementations and reinforced the general need for them.

**Fig 2:**
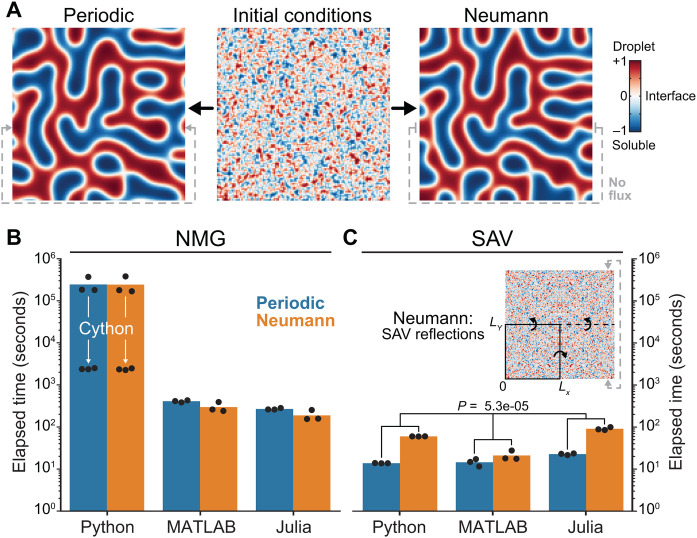
Computing performance depends on the programming language for NMG and the boundary condition for SAV. **(A)** Snapshots of spinodal decomposition when boundary conditions are periodic (left), whereby flux exiting one edge enters on the opposite edge, or Neumann (right) specifying zero flux at the edges. **(B, C)** Runtime performance for NMG (B) and SAV (C) solvers in Python, MATLAB, and Julia for spinodal decompositions initialized with *N* = 3 random mixtures of ±1 chemical states (25:75, 50:50, and 75:25) and either periodic or Neumann boundary conditions. For NMG, the Cython implementation is shown relative to Python. For SAV, Neumann boundary conditions require *X* and *Y* reflections to re-establish periodic boundaries for the expanded spatial domain (C, inset). Boundary conditions in (C) were tested by multiway ANOVA with boundary condition and programming language as factors. All simulations were performed on AMD EPYC 9454 processors (3.81 GHz max, 48 cores per socket, 2 sockets) with up to 500 GB RAM allocated, using up to 16 cores for computation on a 2^7^ × 2^7^ mesh for 2000 time steps (*dt* = 5.5e-6) with *ϵ*_*m*_ = 8.

To compare run times between solvers and among computing environments, we re-simulated ~1% of *t*_*char*_ for three different spinodal initial conditions and two common boundary conditions: i) periodic, in which efflux from one edge is reflected as influx from the opposite edge, and ii) Neumann, or no flux along any edge (Figs 2A and S2, Panels A–B). In Python, a language that many computational systems biologists consider numerically superior to alternatives (i.e., R), we found that NMG was largely unusable without Cython [37] compilation, requiring 10^5^–10^6^ seconds (by extrapolation) for each simulation (Fig 2B). The same implementation of NMG was 1000-fold faster in MATLAB and Julia for both boundary conditions. We attribute these differences to a non-tail recursive function in the NMG solver that is particularly suboptimal for Python [18]. In contrast, SAV implementations yielded similar run times across the computing environments due to shared use of an efficient fast Fourier transform (FFT) algorithm for solving the elliptical equations posed by SAV (Box 3). Also, SAV performance depended on the boundary conditions—unlike NMG, Neumann simulations were handled less efficiently than spinodal decompositions that were periodic (Fig 2C; *P* = 5.3e-05 by multiway ANOVA). Due to the periodicity of the FFT, SAV solutions with Neumann boundary conditions must be solved by reflecting the spatial domain at the boundary, which increases the computations by fourfold (Fig 2C, inset and Materials and Methods). These overall trends persisted for mesh sizes ranging from 2^6^ x 2^6^ to 2^9^ x 2^9^ (S2 Fig, Panels C–D), where we confirmed that NMG and SAV were internally consistent throughout (S2 Fig, Panels E–F). Using results from the largest mesh, we verified that the characteristic length scale of spinodal decomposition grows with *t*_*char*_^~1/3^ (S2 Fig, Panels G–H), consistent with Lifshitz-Slyozov theory [38]. Because of their high initial spatial frequencies, spinodal decompositions are among the most difficult Cahn-Hilliard solutions, making them useful to discern whether the time step is appropriate for a given mesh size.

### Long-term Cahn-Hilliard simulations define universal parameters for condensate stability

**Fig 3:**
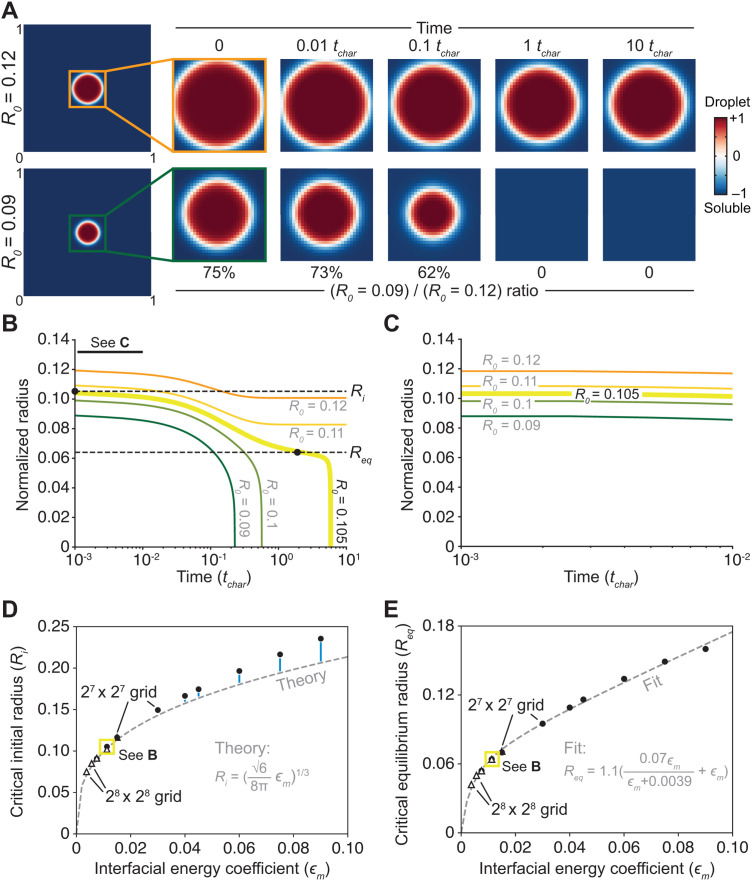
Multithreaded Cahn-Hilliard simulations define the relationship between *ϵ*_*m*_ and critical droplet radii. **(A)** Simulated dynamics for two normalized initial droplet radii (*R*_*0*_) on a 2^8^ x 2^8^ mesh (*L*_*X*_ = *L*_*Y*_ = 1) for 400,000 time steps (*dt* = 2.5e-5) and *ϵ*_*m*=12_ ≈ 0.011. The interface between the circular droplet and soluble phase was initialized to the hyperbolic tangent for an infinite interface (Box 2 and Materials and Methods). The proportion of *R*_*0*_ = 0.09 relative to *R*_*0*_ = 0.12 is shown for each characteristic time point (*t*_*char*_). **(B)** Scan of *R*_*0*_ to determine the critical initial radius (*R*_*i*_) and the critical equilibrium radius (*R*_*eq*_) for a given *ϵ*_*m*_. *R*_*i*_ was calculated at *t* = 0 between the largest *R*_*0*_ that dissolves away (yellow) and the smallest *R*_*0*_ that persists (marigold). *R*_*eq*_ was estimated from the inflection point of the largest dissolving droplet (Materials and Methods)*.* **(C)** Enlargement of the first 0.1% of the time simulated in (B). **(D, E)** Calculation of *R*_*i*_ (D) and *R*_*eq*_ (E) as in (B*)* from *ϵ*_*m*=4_ on a 2^8^ x 2^8^ mesh (= 0.0037) to *ϵ*_*m*=48_ on a 2^7^ x 2^7^ mesh (= 0.0901). Calculations for *ϵ*_*m*=8,12,16_ on a 2^8^ x 2^8^ mesh (triangles) were repeated for *ϵ*_*m*=4,6,8_ on a 2^7^ x 2^7^ mesh (circles) to confirm overlap. In (D), calculations are compared to a prior theory [39] (inset) relating *R*_*i*_ and *ϵ*_*m*_; systematic deviations are indicated in blue. In (E), calculations are compared to a hyperbolic-to-linear fit (inset); goodness-of-fit was 0.997 for 0.0037 ≤ *ϵ*_*m*_ ≤ 0.0901; extrapolations are not recommended. The approximate condition in (B) is highlighted (yellow box).

The Cahn-Hilliard equation is handled non-dimensionally, but it formalizes concrete relationships between space, time, concentration, and diffusivity that are frameable in absolute terms (Boxes 1 and 2). The one unknown parameter is the interfacial energy coefficient defining the transition distance between phases (*ϵ*_*m*_; Eqs 7 and 8), raising the practical question of how to constrain it for a specific biological system. We considered exploiting the criticality of Cahn-Hilliard fluids, whereby small phase-separated droplets dissolve into the bulk when there is not enough mass to retain them at a given surface tension (Fig 3A). For any *ϵ*_*m*_, there exists a critical droplet size below which droplets will not persist over time (S1 Movie). The radius of this droplet may be defined in terms of its critical initial radius (*R*_*i*_) at *t* = 0 or its critical equilibrium radius (*R*_*eq*_) as *t* → ∞. Although there is theory linking *ϵ*_*m*_ to *R*_*i*_ under prescribed assumptions [39], the relationship between *ϵ*_*m*_ and *R*_*eq*_ is unknown but important because *R*_*eq*_ is estimable by experiments.

Defining critical radii requires long simulations, because droplets near the bifurcation often do not dissolve until one *t*_*char*_ or more has passed (Fig 3A). We systematically explored a range of starting non-dimensional radii (*R*_*0*_, which is normalized to the overall spatial domain) and simulated up to 10*t*_*char*_ with the stable NMG–SAV solvers by multithreading individual runs on a supercomputer. *R*_*i*_ was defined as the midpoint between the smallest *R*_*0*_ that did not dissolve and the largest *R*_*0*_ that did, whereas *R*_*eq*_ was approximated at the inflection point in time when the largest dissolving *R*_*0*_ starts to vanish (Fig 3B). Notably, none of these changes were evident with the first 1% of *t*_*char*_ (Fig 3C), reinforcing the need for numerically stable, efficient, and accurate solvers as provided here (Fig 1). Solutions for *R*_*i*_ and *R*_*eq*_ at a specific *ϵ*_*m*_ did not change if the starting radius was located off center in the spatial domain or split across the domain when boundary conditions were periodic (S3 Fig, Panels A–B). Thus, *R*_*i*_ and *R*_*eq*_ are fundamentally linked to the *ϵ*_*m*_ of a Cahn-Hilliard system.

By repeating *R*_*0*_ sweeps across many *ϵ*_*m*_ values, we numerically defined the general relationship between *ϵ*_*m*_ and *R*_*i*_ or *R*_*eq*_ on a 2^7^ x 2^7^-element mesh (Fig 3D–3E). For small *ϵ*_*m*_ values, 2^7^ elements were insufficient to specify the interfacial transition accurately (*m* ≥ 4 mesh points is recommended). Therefore, a 2^8^ x 2^8^-element mesh was substituted with enough overlap at intermediate *ϵ*_*m*_ values to confirm that results were identical. We observed excellent concordance between an approximation relating *R*_*i*_ and *ϵ*_*m*_ when *ϵ*_*m*_ values were small (Fig 3D), corroborating the underlying assumptions of this derivation [39]. At large *ϵ*_*m*_ values, however, there was a systematic underestimate by the theory, consistent with the omission of higher-order terms in its derivation. For *R*_*eq*_, we sought an empirical regression that was simple and accurate, finding that a three-parameter hyperbolic-to-linear equation [40] was best among alternatives (Figs 3E and S3, Panels C–F). Together, these relationships provide a reference for fully parameterizing any Cahn-Hilliard system given *R*_*i*_ or *R*_*eq*_.

### Cahn-Hilliard abstraction of the chromosomal passenger complex

**Fig 4:**
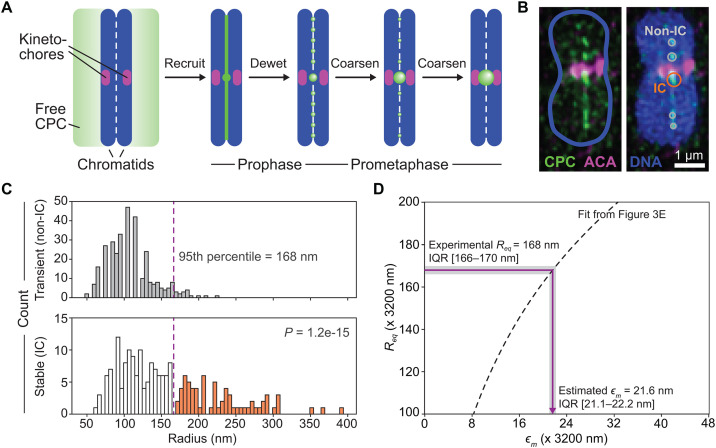
Chromatin-bound foci outside the inner centromere (IC) define a critical equilibrium radius (*R*_*eq*_) for the chromosomal passenger complex (CPC). **(A)** CPC recruitment to the IC during prophase and prometaphase. Freely diffusible CPC (green) and chromatin bound CPC is recruited strongly to the IC between kinetochores (magenta) and less so between sister chromatids. During late prophase, CPC dewets into discrete foci, which coarsen until all CPC is IC-localized by prometaphase. **(B)** Immunofluorescence illustration of CPC dewetting on a HeLa chromosome stained for AURKB (a CPC subunit; green), anti-centromeric antigen (ACA; magenta), and DAPI to label DNA (blue). ACA labeling of kinetochores distinguishes IC foci (orange) and non-IC foci (gray) of CPC during quantification (Materials and Methods). **(C)** Histogram of non-IC foci (gray) and IC foci (white and orange) quantified by idealized circular radius from *N* = 221 chromosomes in 10 HeLa cells. The 95th percentile of the non-IC droplet size distribution defining *R*_*eq*_ is shown. The distribution of non-IC foci (*N* = 321) and IC foci (*N* = 195) were compared by KS test. **(D)** Estimation of *ϵ*_*m*_ from measured *R*_*eq*_ using the hyperbolic-to-linear regression (Fig 3E) scaled for a 3200-nm physical spatial domain. The interquartile range (IQR) was calculated by 50% subsampling of *N* = 10 HeLa cells for 100 iterations without replacement and propagating to the *ϵ*_*m*_ estimate.

We next sought a biological setting to apply the NMG–SAV solvers and Cahn-Hilliard constraints. In cells, biomolecular condensates form in the nucleus upon multivalent interactions with DNA [10]. One highly dynamic example of chromatin binding involves the chromosomal passenger complex (CPC), a multi-protein assembly that is recruited to mitotic chromosomes between prophase and metaphase [21]. CPC binding occurs through two chromatin modifications that overlap at the inner centromere between kinetochores of the sister chromatids (Fig 4A). One of the modifications (phospho-histone H3 Thr3, pH3T3) emanates from a kinase bound to cohesin, which holds together the sister-chromatid arms. The monovalent affinity [41] of the pH3T3-CPC interaction is strong enough to recruit some CPC between sister chromatids in prophase, but only CPC recruited to the inner centromere, where there is a partially overlapping second histone phosphorylation, persists into metaphase [42].

Previously, Trivedi et al. [22] reported that inner centromere-bound CPC reaches local concentrations high enough for its intrinsically disordered region (IDR) to form biomolecular condensates during mitosis. This claim was recently called into question [23] based on new experiments involving how CPC foci form and dissolve at the inner centromere. We noted that the dynamics of the CPC from prophase to metaphase (idealized in Fig 4A) resembled two properties of a Cahn-Hilliard system. First, a spinodal dewetting breaks up monovalently bound CPC into smaller foci during prophase. Then, coarsening occurs during prometaphase, in which smaller CPC foci disappear as the larger inner centromere focus grows to become singular at the onset of metaphase. The µm length scale of changes (Fig 4B), the short ~20-minute duration of (pro)metaphase [43], and the diffusivity of chromatin [44] are all self-consistent within a Cahn-Hilliard framework (Box 2) without further elaborations [45]. Therefore, we modeled chromatin-bound CPC as a Cahn-Hilliard chemical state (+1; soluble CPC = –1) that could be tracked and tested by experiment.

We began with measurements in HeLa cells to build off earlier work [22,23,42] and later examined the generality of the results using nontransformed cells of a different lineage. CPC binding was visualized on chromosomes by immunostaining mitotic spreads prepared after timed G2 release (Materials and Methods). We stained chromosomes for AURKB (a subunit of the CPC) along with anti-centromeric antigen (ACA) to localize kinetochores and the inner centromere (Fig 4B). Most spreads showed CPC localized exclusively to the inner centromere (consistent with the rightmost cartoon of Fig 4A), but a fraction of cells exhibited multi-focal staining between sister chromatids (Fig 4B). The CPC characteristics of chromosomes in these multi-focal spreads were used to parameterize the Cahn-Hilliard equation and, eventually, to test it by an independent set of measurements.

We constrained the interfacial energy coefficient by leveraging the general relationship between *ϵ*_*m*_ and *R*_*eq*_ (Fig 3E). We quantified the area of CPC foci from diffraction-limited confocal images that were deconvolved and z-projected (Materials and Methods), converting the area to an equivalent radius for a circle (Fig 4B). Recognizing that CPC foci at the inner centromere persist and those elsewhere do not (Fig 4A), we stratified the measurements and noted that inner centromere foci had significantly larger radii (Fig 4C; *P* = 1.2e-15 by Kolmogorov–Smirnov (KS) test). The partial size overlap with other foci was presumably because these inner centromere foci were still growing at the experimental endpoint (Fig 4A, middle), making it difficult for this distribution to define *R*_*eq*_. Therefore, we approached *R*_*eq*_ by instead using the distribution of non-inner centromere foci, all of which ultimately dissolve. Taking the upper 95th percentile of non-inner centromere foci, we estimated *R*_*eq*_ = 168 ± 2 nm for HeLa cells. We defined a square domain based on the ~ 95th percentile of measured chromosome arms (2 x 3.2 µm = 6.4 µm; S4 Fig, Panel A) and used the relationship between *ϵ*_*m*_ and *R*_*eq*_ to define *ϵ*_*m*_ = 21.6 ± 0.6 nm (Fig 4D). Such a distance could be spanned by the ~ 32-nm single alpha-helical “dogleash” in between the chromatin-bound subunits of the CPC and kinase subunit, allowing the kinase to act inside or outside a putative condensate (S4 Fig, Panel B) [46].

**Fig 5:**
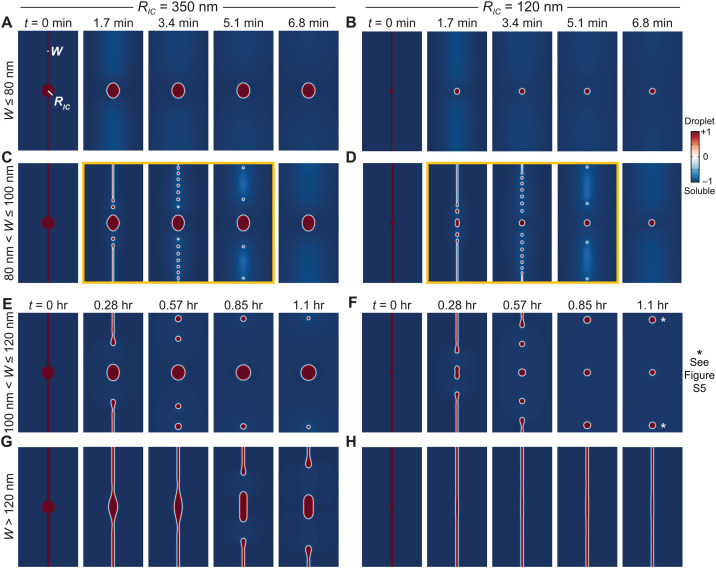
Cahn-Hilliard simulations predict extended multi-droplet regimes for physical values of CPC inner centromere radius (*R*_*IC*_) and pH3T3 width (*W*). **(A–D)** Dynamics at 1, 2, 3, and 4% *t*_*char*_ (corresponding to the indicated time in minutes based on chromatin diffusivity and mesh size; Eq 11) for *W* = 60 nm (A–B) *or* 90 nm (C–D) and *R*_*IC*_ = 350 nm (A, C) or 120 nm (B, D). Sustained droplets are highlighted in (C) and (D) (yellow). **(E–H)** Dynamics at 10, 20, 30, and 40% *t*_*char*_ for *W* = 120 nm (E–F) or 140 nm (G–H) and *R*_*IC*_ = 350 nm (E, G) or 120 nm (F, H). Sustained droplets (asterisks) are described further in S5 Fig. Droplet patterns are representative for the intervals of *W* listed on the left. All CPC simulations were performed on Intel Xeon Gold 6248 processors (2.50 GHz base, 20 cores per socket, 2 sockets) with 200 GB RAM allocated, on a 2^9^ x 2^9^ mesh (*L*_*X*_ = *L*_*Y*_ = 2) for 26,214 (A–D) or 262,144 (E–H) time steps (*dt* = 1.53e-6).

With a fully constrained system, we next examined the geometric requirements for a CPC “initial condition” at prophase (Fig 4A). Monovalent CPC recruitment was cast as a rectangle spanning the vertical domain with a prescribed width (*W*), upon which we overlaid recruitment to the inner centromere as a central circle with a radius of *R*_*IC*_ (Fig 5A). Initializing CPC as a rectangular crosshair [47] quickly coarsened to a round droplet and yielded similar results to *R*_*IC*_ (S2 Movie). We initialized Cahn-Hilliard simulations for large and small *R*_*IC*_ based on prior measurements of CPC foci at the inner centromere, and finer intervals of *W* were guided by CPC radii detected elsewhere (Fig 4C). Dynamics over the first 0.04*t*_*char*_ ≈ 7 minutes were considered realistic for prometaphase, but slowly evolving initial conditions were followed for nearly 70 minutes to define limiting behavior. The goal was to identify regimes of *R*_*IC*_ and *W* in which dewetting then ripening was observable on the time scale of prometaphase.

Overall, we found that the emergent properties of the system depended disproportionately on *W*, with *R*_*IC*_ only affecting the final size of the central droplet except when *W* was exceedingly large (compare Fig 5A–5D to Fig 5E–5H). When *W* < 80 nm, axial droplets emerged and disappeared within 13 seconds, which would be too fast to see in fixed chromosomes (Figs 4B and 5A–5B). However, when *W* was increased to 80–100 nm, droplets persisted for several minutes before dissolving, providing enough time to capture them experimentally (Figs 4B and 5C–5D). This interval of *W* is notable because the cohesin holocomplex is 64 ± 7 nm long [48], and the pH3T3 kinase is tethered to cohesin by an extended linker on the N terminus that is likely unstructured [49,50]. If this linker behaved as a random coil [51], its radius of gyration would be ~ 5 nm and thus add as much as ~20 nm to the effective width of cohesin for monovalent CPC recruitment, placing *W* in the 80–100 nm interval (S5 Fig, Panel A).

Beyond 100 nm, the dynamics of the system were considerably slower. When *W* > 120 nm, the stripe was too stable and no droplets formed for over one hour (Fig 5G–5H). Intriguingly, between 100 nm and 120 nm, a fourth regime slowly emerged, in which multiple droplets persisted for tens of minutes along the vertical axis (Fig 5E–5F). This situation is not expected to arise ordinarily, but we identified one perturbation in the literature that may evoke it. Gascoigne et al. [52] engineered HeLa cells to assemble nascent kinetochores ectopically throughout a chromosome, which should enlarge *W* by expanding the domain of inner centromere-like CPC recruitment (S5 Fig, Panel B). Revisiting the authors’ original images of CPC localization in metaphase-arrested cells, we identified lines of multiple foci on chromosomes with ectopic kinetochores, which were absent from controls (S5 Fig, Panels C–D). Although not a formal prediction of the Cahn-Hilliard model, this observation built confidence in the physical dimensions of the system and our parameterization of *ϵ*_*m*_.

### Cahn-Hilliard models accurately predict spacing of CPC foci in multiple cellular contexts

**Fig 6:**
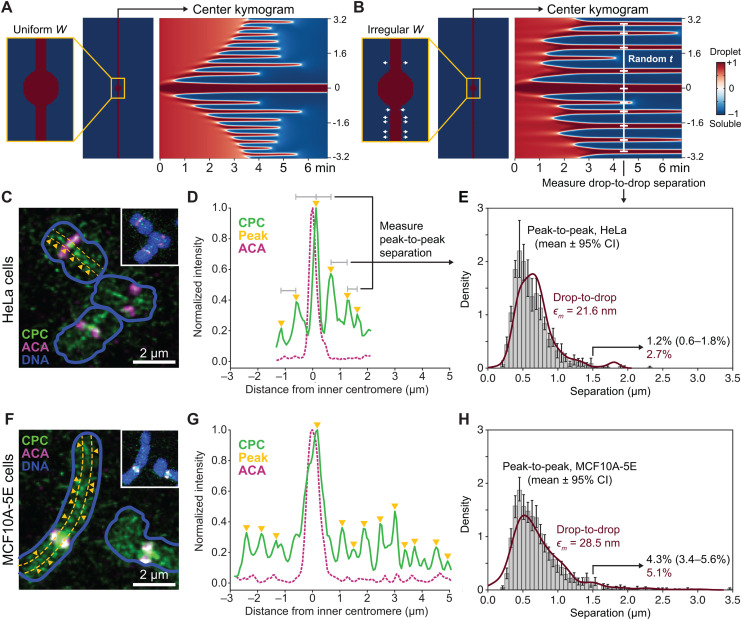
Chromatin-bound CPC behaves as a phase-separated Cahn-Hilliard fluid. **(A, B)** Kymogram illustration of central droplets for 6.8 minutes after initialization with *R*_*IC*_ = 150 nm and *W* = 90 nm (A) or 90 ± 10 nm (B, white arrows). Drop-to-drop distances were quantified at randomly selected times *t*. **(C)** Immunofluorescence staining of dewetted CPC on HeLa chromosomes after G2 release for 10 minutes. **(D)** Normalized intensity of AURKB (CPC) and ACA for the HeLa chromosome in (C). **(E)** Comparison of bootstrapped peak-to-peak separation of CPC foci in HeLa cells (gray histogram) with drop-to-drop distances from Cahn-Hilliard simulations with *ϵ*_*m*_ = 21.6 nm (red line). **(F)** Immunofluorescence staining of dewetted CPC on MCF10A-5E chromosomes after G2 release for 70 minutes. **(G)** Normalized intensity of AURKB (CPC) and ACA for the MCF10A-5E chromosome in (F). **(H)** Comparison of bootstrapped peak-to-peak separation of CPC foci in MCF10A-5E cells (gray histogram) with drop-to-drop distances from Cahn-Hilliard simulations with *ϵ*_*m*_ = 28.5nm (red line). For (C, F), cells were stained for AURKB (a CPC subunit; green), anti-centromeric antigen (ACA; magenta), and DAPI to label DNA (blue). A 300-nm-thick axial line scan (dotted yellow line) was used to identify peaks of AURKB foci (yellow triangles; Materials and Methods). For (D, G), normalized fluorescence intensities are shown relative to distance from the inner centromere based on the peak ACA location centered at zero. Peaks of AURKB foci (yellow triangles) were identified with a computational algorithm (Materials and Methods) and quantified. For (E, H), data are shown as the median ± 95% bootstrapped confidence interval (CI) from *N* = 221 chromosomes in 10 cells (E) or 216 chromosomes in 50 cells (H). The fraction of predicted (red) and measured (black) separations greater than 1.5 µm is indicated.

We originally calibrated *ϵ*_*m*_ to the size distribution of bound CPC foci (Fig 4C–4D). Since this calibration included no information about the location of foci along a HeLa chromosome, we reasoned that their spatial distribution would serve as an independent prediction of the resulting model. We focused on the multi-droplet regime based on prior structural arguments [48–51] supporting feasibility (*W* = 80–100 nm; Fig 5C–5D). In the idealized case, transient droplets appear and disappear with perfect symmetry on either side of the inner centromere (Fig 6A). Biologically, however, there is uncertainty about the precise location of the H3T3 kinase (S5 Fig, Panel A), and pH3T3 marks are spatially rougher [53]. Therefore, we sampled *W* from a normal distribution (*µ* = 90 nm, *σ* = 10 nm) for each mesh row at its midpoint, which yielded an irregular pattern of longer-lived droplets (Fig 6B and S3 Movie). Assuming that the immunofluorescence images were random snapshots of this dynamic, we built a gallery of Cahn-Hilliard movies for 10 *R*_*IC*_ values ranging from 50–350 nm (Fig 4C, bottom) and randomly sampled time points to build a predicted distribution of droplet-to-droplet distances (Fig 6B and Materials and Methods). In parallel, we revisited the HeLa images and performed 300-nm-thick line scans of CPC–ACA staining between sister chromatids (Fig 6C), identifying local maxima with a peak-finding algorithm (Fig 6D and Materials and Methods). Using ACA to orient the inner centromere, we quantified peak-to-peak distances between CPC foci and estimated uncertainty in distance frequency by bootstrapping. The resulting histogram of measured peak-to-peak distances (Fig 6D) was directly comparable to the density of drop-to-drop distances predicted earlier by the Cahn-Hilliard model (Fig 6B).

We found that Cahn-Hilliard predictions of spatial frequency were nearly superimposable with CPC measurements in HeLa cells (Fig 6E). Separation distances of 500–600 nm were most common, and the right-tailed distribution decreased sharply with distance such that spacings were rare above 1 µm and almost nonexistent above 1.5 µm. By physically constraining a diffuse interface (*ϵ*_*m*_; Fig 4C–4D) and an initial geometry (*W* and *R*_*IC*_; Fig 5C–5D), the Cahn-Hilliard equation yields dynamics (Fig 6B) and length scales (Fig 6E) that are fully consistent with CPC biology without any further refinements.

Is *ϵ*_*m*_ a universal or context-specific property of the CPC? To address this question, we pivoted from HeLa cells, a highly aneuploid and divergent [54] cervical cancer line, to the 5E subclone [55] of MCF10A cells—a pseudo-diploid, non-cancerous [56] breast epithelial line. Compared to HeLa, MCF10A-5E cells required much more time after G2 release to enter mitosis (Materials and Methods). Further, CPC foci were most evident earlier in prophase, when chromosomes were not as fully condensed and thus more elongated (*P* = 7.7e-48 by KS test; Figs 6F and S4, Panel A). We repeated the *R*_*eq*_ estimation for CPC as before and found that *ϵ*_*m*_ for MCF10A-5E cells was ~ 30% larger than for HeLa cells (28.5 nm ± 1.1 nm; S6 Fig, Panels A–B). The increase may relate to overall chromatin compaction, specific binding modes, or differences in CPC modification state (see Discussion), but regardless the MCF10A-5E data indicate that the *ϵ*_*m*_ for CPC is not fixed.

The larger *ϵ*_*m*_ of MCF10A-5E simulations did not qualitatively impact whether multiple droplets formed over the same range of initializations (*W* = 80–100 nm; *R*_*IC*_ = 50–350 nm), but it did alter the droplet spacing. For the same predicted median spacing of ~640 nm, the predicted variance for MCF10A-5E droplets was greater, especially on the right tail of the distribution (S6 Fig, Panel C). We repeated the line-scan and peak calls for CPC foci on MCF10A-5E chromosomes and observed the same exaggerated right tail compared to HeLa (Figs 6F–6G and S6, Panel D). When Cahn-Hilliard predictions and MCF10A-5E measurements were directly compared, there was excellent agreement, including more-frequent events in the 1–1.5 µm range and instances that extended beyond 1.5 µm (Fig 6H). We conclude that the CPC behaves as a simple Cahn-Hilliard fluid early in mitosis. More generally, the numerical methods gathered here enable a complementary validation strategy [23] for biomolecular condensates that is purely mathematical.

**Box 1. Chemical states and energy functions in a Cahn-Hilliard system.**

**Fig 7:**
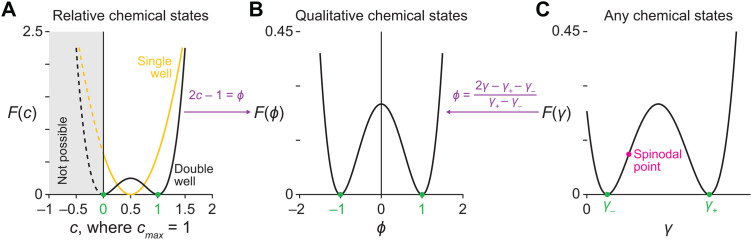
The Cahn-Hilliard double-well free energy function (*F*) accommodates differences in location and scale. **(A)** Comparison of a single-well function (yellow, with *c*_*avg*_ = 0.5) and a double-well function (black, stable states at 0 and 1 in green). **(B)** The double-well function of (A) is made symmetric about the y-axis with linear shifts and scaling (purple). **(C)** Any pair of chemical states (*γ*_*–*_ and *γ*_*+*_ in green) is similarly shifted and scaled to **(B)**. The spinodal point (magenta) is constrained.


The Cahn-Hilliard equation models the dynamics of dimensionless chemical states. It is straightforward to convert a real chemical concentration (*rcc* [=] mass/length^3^) to a dimensionless relative chemical state (*c* [=] dimensionless) by normalizing to the concentration of the species at saturation (*rcc*_*max*_ [=] mass/length^3^): *c* = *rcc* / *rcc*_*max*_. In a purely diffusive system, the free energy (*F*) of *c* from Flory–Huggins solution theory is approximated by a parabola [57] with a minimum at the well-mixed average (*c*_*avg*_): $F(c)=\alpha {\left(c-c_{avg}\right)}^{\text{2}}+\beta$. *α* and *β* specify the units and reference state for *F*, but they do not impact the equilibrium solution of minimum free energy (and thus zero chemical potential) where *dF*/*dc* = 0 (Fig 7A, yellow). To simplify the presentation and help with later scaling arguments, we keep *F* dimensionless by normalizing *F* by *k*_*B*_*T* (where *k*_*B*_ is the Boltzmann constant and *T* is absolute temperature) and take *α* = 1, *β* = 0.

For a Cahn-Hilliard two-phase system [17], an alternative double-well free energy function is used to approximate a Flory–Huggins solution with interacting phases (also known as the regular solution theory; Fig 7A, black), which we begin to introduce here in terms of stable minima at *c* = 0, 1 and *α* = 1, *β* = 0 (Fig 7A, green):

$$\begin{array}{c} F(c)=c^{\text{2}}(c-\text{1}{)}^{\text{2}} \end{array}$$ (1)

Note that any pair of stable minima can be rescaled to fall on a [0,1] interval by linearly shifting the minimum and dividing by the range. For stable minima *γ* = *γ*_*–*_, *γ*_*+*_:

$$\begin{array}{c} =\frac{\gamma -\gamma_{-}}{\gamma_{+}-\gamma_{-}} \end{array}$$ (2)

For mathematical convenience, it is advantageous to convert the double-well function of Eq 1 to a pair of qualitative chemical states at *ϕ* = –1, 1 by linearly translating and scaling (Fig 7B, purple):

$$\begin{array}{c} \phi =\text{2}c-\text{1} \end{array}$$ (3)

to yield:

$$\begin{array}{c} F(\phi )=(\phi +\text{1}{)}^{\text{2}}(\phi -\text{1}{)}^{\text{2}}={\left(\phi^{\text{2}}-\text{1}\right)}^{\text{2}} \end{array}$$ (4)

This symmetric double-well function of Fig 7B is found most often in the Cahn-Hilliard literature, but system behavior is alternatively described according to its spinodal point—the minimum concentration at which two phases stably coexist. The spinodal point is equivalent to the left inflection point of the free energy. Differentiating Eq 4 twice and solving for the negative root yields:

$$\begin{array}{c} \phi_{spinodal}=-\frac{\sqrt{\text{3}}}{\text{3}} \end{array}$$ (5)

The spinodal point generalizes to any concentration by taking Eq 5, converting *ϕ* to *c* and then *c* to *γ* (Eqs 2 and 3; Fig 7C):

$$\begin{array}{c} \gamma_{spinodal}=\frac{\text{3}-\sqrt{\text{3}}}{\text{6}}\left(\gamma_{+}-\gamma_{-}\right)+\gamma_{-} \end{array}$$ (6)

Thus, for a symmetric double-well function, it is not possible to set *γ*_*–*_, *γ*_*+*_, and *γ*_*spinodal*_ independently—two concentrations define the third (Fig 7C). This property is useful for determining whether a free energy function such as Eq 1 is appropriate for a phase-separated system.

**Box 2. Dimensional analysis of the Cahn-Hilliard equation.**

For qualitative chemical states (*ϕ* = –1, 1 [=] dimensionless; Box 1), the Cahn-Hilliard equation is succinctly defined as:

$$\begin{array}{c} \frac{\partial \phi }{\partial t}=D\nabla^{\text{2}}\mu =D\nabla^{\text{2}}\left(\frac{dF}{d\phi }-\epsilon_{m}^{\text{2}}\nabla^{\text{2}}\phi \right) \end{array}$$ (7)

where *D* is diffusivity ([=] length^2^/time), $\nabla^{\text{2}}$ is the Laplacian, *µ* is a generalized chemical potential ([=] energy nondimensionalized by *k*_*B*_*T*; Box 1), $\frac{dF}{d\phi }$ is the first derivative for Eq 4 ([=] nondimensionalized energy), and $\epsilon_{m}$ is the “interfacial energy coefficient”, a transition distance ([=] length•{nondimensionalized energy}^1/2^) operationally defined as:

$$\begin{array}{c} \epsilon_{m}=\frac{mh}{\text{2}\sqrt{\text{2}}{tanh}^{-\text{1}}\text{0}.\text{9}\;} \end{array}$$ (8)

where *h* is the fractional mesh size of the spatial domain (*h* = $\frac{L}{{\text{2}}^{\text{6}}}$, $\frac{L}{{\text{2}}^{\text{7}}}$, $\frac{L}{{\text{2}}^{\text{8}}}$, etc. where *L* is the size of the spatial domain [=] length) and *m* is the number of mesh points over which the interface exists ([=] dimensionless). (The $\text{2}\sqrt{\text{2}}{tanh}^{-\text{1}}\text{0}.\text{9}\;\approx \;\text{4}$ normalization arises from the equilibrium one-dimensional solution [58] for an infinite interface, $\phi (x)=tanh\left(\frac{x}{\sqrt{\text{2}}\epsilon_{m}}\right)$, when considered over the range of *ϕ* = –0.9 to *ϕ* = 0.9. Thus, $\epsilon_{\text{4}}\;\approx \;h$ and $\epsilon_{\text{8}}\;\approx \;\text{2}h$.) In Eq 7, $\frac{dF}{d\phi }\;$in *µ* captures departures of *F*(*ϕ*) from its thermodynamic minima (Fig 7B), and the $-\epsilon_{m}^{\text{2}}\nabla^{\text{2}}\phi$ term generalizes *µ* to approximate interfacial surface energy for the diffuse interface between the two chemical states [17]. Solving for an unknown $\epsilon_{m}$ is equivalent to defining *m* for a given *h*.

The generalized chemical potential *µ* is defined in terms of *ϕ* and normalized in such a way that its second-order changes occur on the same order of magnitude as changes in *ϕ* itself at all times (Box 1). Therefore, the effect of these field variables cancels and by scaling analysis:

$$\begin{array}{c} t_{char}=\frac{L_{char}^{\text{2}}}{D} \end{array}$$ (9)

The solvers in this work set *D* = 1 for the general solution case. The actual physical dimensions of *D*, $\epsilon_{m}$, and *h* set different characteristic times (*t*_*char*_) depending on what length scale tha*t* diffusion is operating. For one-dimensional diffusion of *ϕ* across the operational interface in Eq 8:

$$\begin{array}{c} t_{char}=\frac{{\text{2}\epsilon }_{m}^{\text{2}}}{D} \end{array}$$ (10)

and for two-dimensional diffusion in the overall system:

$$\begin{array}{c} t_{char}=\frac{\text{2}{\left(\frac{mh}{\text{2}}\right)}^{\text{2}}+\text{2}{\left(\frac{mh}{\text{2}}\right)}^{\text{2}}}{D}=\frac{(mh{)}^{\text{2}}}{D} \end{array}$$ (11)

Taking a 3.2 µm x 3.2 µm patch of chromatin with *D* = 10^-3^ µm^2^/second [44], a 2^8^ = 256-element mesh, and $\epsilon_{\text{4}}\;\approx \;h=\text{3}.\text{2}\;\mu m/{\text{2}}^{\text{8}}=\;\text{0}.\text{0}\text{1}\text{2}\text{5}\;\mu m$ yields *t*_*char*_ = 0.312 seconds for diffusion across the interface, which limits *t*he time step of the solver, and *t*_*char*_ = 2.84 hours for diffusion across the system, which defines how long the solver mus*t* run. (Note that for a same-sized patch of cytoplasm with *D* ~ 10 µm^2^/second [59], diffusion across the system would occur with *t*_*char*_ = 1.02 seconds.) Reciprocally, fixing a timeframe of interest defines a charac*t*eristic length scale: 10 minutes of chromatin diffusion will influence distances of biomolecular condensates on the order of 775 nm.

**Box 3. Nonlinear multigrid (NMG) and scalar auxiliary variable (SAV) approaches to solving the Cahn-Hilliard equation.**

In the NMG approach, spatial derivatives are cast as centered-difference approximations, and the method uses a semi-implicit time discretization with a nonlinear convex-splitting scheme for the free energy (Eqs 1 and 4). This combined scheme ensures that a discrete version of the free energy is always decreasing and thus stable with time. NMG solves the nonlinear discrete equations at the implicit time level using the full approximation storage (FAS) multigrid method [60]. The discrete system is solved iteratively on a hierarchy of square 2^*n*^ meshes (*n* = 6–9) that are obtained by successively coarsening the original mesh by factors of two in each direction. Smoothing is used to remove high frequencies and ensure accurate approximations of the full solution on the coarser meshes, as opposed to the error in the linear multigrid method. Coarse grid corrections are interpolated back to the fine grid and the corrected solution is smoothed. The nonlinearity is handled using a local linearization with Newton’s method and a pointwise Gauss–Seidel relaxation scheme is used as the smoother [18,61]. This process is repeated recursively until convergence at each time step. NMG is more stable and efficient than standard Newton-type methods that rely on global linearizations (Fig 1) or algorithms that treat the nonlinear terms as forcing functions [61].

For SAV, a new scalar variable is introduced in terms of the integrated non-gradient part of the free energy (*F*(*ϕ*) of Eq 7). Eq 7 is then appended with an additional ordinary differential equation for this scalar variable [35]. Straightforward time discretizations of the reformulated system lead to decoupled linear, elliptic partial differential equations with constant coefficients. The decoupled equations are efficiently solved using the discrete Fourier transform [62] for appropriate boundary conditions (periodic or Neumann). Like NMG, SAV is also energy stable in that the reformulated discrete energy, containing the scalar “auxiliary” variable, does not increase with time. To ensure that the reformulated discrete energy is consistent with the original system energy, a relaxation scheme is used to penalize discrepancies that may arise between the reformulated and original energies. For further details, see [63] and Materials and Methods.

## Discussion

The Cahn-Hilliard equation emerged from chemical physics but has found application in many areas of biology [1–6,8,9,17]. The refined NMG and SAV solvers presented in this work bring Cahn-Hilliard modeling closer to the realm of systems biology. The two numerical methods are not redundant but rather complementary. NMG is a more-established approach [61] that uses its own defined functions recursively to solve the Cahn-Hilliard equation within a specified error tolerance. By checking against the Cahn-Hilliard equation at each time step, NMG acts as a standalone solver. The newer SAV approach [35] changes the problem into a set of elliptical equations that are efficiently solved by a separate algorithm [62] for discrete Fourier transforms. SAV is generally faster than NMG; however, care must be taken to ensure that SAV stays true to the actual Cahn-Hilliard solution. Our SAV implementation incorporates relaxation [63] to help with problematic initial conditions, like spinodal decomposition, and solution drift with very large time steps. When SAV performance is uncertain, NMG serves as a useful crosscheck for self-consistency.

We used Cahn-Hilliard simulations as a test of dewetting–coarsening mechanisms that might evolve localization of the CPC and found that predictions were highly supportive of phase separation with *ϵ*_*m*_ specifying the diffuse interface width. By operating in physical units, parameter estimates were directly relatable to molecular entities. The linker subunit of the CPC has a ~ 32-nm long alpha helix [46] preceded by an intrinsically disordered region that adds 5–14 nm depending on its phosphorylation state [64]. Interestingly, neither of these regions is necessary for the CPC to form a condensate [22], raising the possibility that they may instead contribute to the magnitude of *ϵ*_*m*_. Although we did not detect cell-specific differences in overall phosphorylation of the CPC linker, another subunit was differentially altered, and there was a consistent 2–3-fold difference in overall abundance that might alternatively impact *ϵ*_*m*_ indirectly (S6 Fig, Panels E–F). A long-term interest is to extend this framework to other phase-separated systems warranting compartmental models [65,66].

Condensation of the CPC may be evolutionarily advantageous for the robustness it endows. In a Cahn-Hilliard system, larger droplets fuse with smaller droplets (or pull mass from them) by Ostwald ripening until one droplet or zero droplets remains. The phenomenon acts as an analog-to-digital converter, which filters smaller, off-target droplets outside of the inner centromere. From this perspective, the CPC may employ two histone-binding activities emanating from orthogonal axes to ensure that the inner centromere generates a large enough *R*_*IC*_ so that it reliably dominates over alternatives [21].

A limitation of the standard Cahn-Hilliard equation (and, by extension, the NMG–SAV solvers) is the lack of source and sink terms for synthesis and degradation over time. Source–sink terms can be appended to the governing equation along with feedback [3], but given the number of possible configurations we opted for simplicity in the software packages. Constant mass is a reasonable assumption for the ~ 6-min dynamics of the CPC, although we recognize it may be less satisfactory for systems that evolve more slowly with time. Other biomolecular condensates that are active or reactive during the time scale of interest require modeling formalisms beyond the Cahn-Hilliard equation [45].

Biomolecular condensates are integrated or involved in many steps of cellular regulation. The properties of isolated condensates are well documented [10–12], but condensation is also a mechanism for conferring switch-like behavior to signaling pathways [67,68]. Stitching Cahn-Hilliard dynamics into biochemical reaction-diffusion networks with these modern solvers is a next frontier for systems-biology modeling.

## Materials and methods

### NMG solver

We refactored the NMG algorithm of Lee et al. [18] by hierarchically organizing local and global functions, integrating global functions with the SAV solver, and clarifying variable nomenclature usage. ‘CahnHilliard_NMG’ takes an initial chemical state field (*ɸ*_0_; Box 1) provided by the user or created by ‘ch_initialization’ and uses ‘nmg_solver’ with recursive calls to ‘nmg_vcycle’ that solve for *ɸ* at each time step *dt*. ‘CahnHilliard_NMG’ handles the following simulation parameters with the variable name and default value in parentheses: number of time iterations (t_iter = 1e3), the size of the time step (dt = 2.5e-5), the number of solver iterations per time step (solver_iter = 1e4), the absolute solver tolerance per time step (tol = 1e-5), the number of mesh points *m* for *ϵ*_*m*_ (m = 8), the boundary conditions (boundary = ‘periodic’), the number of smoothing relaxations done at the start and end of each coarsening–interpolation cycle (c_relax = 2), the domain size in terms of right and left coordinates of x and y (domain = [1 0 1 0]; note that [2 0 2 0] was used for the CPC simulations in Figs 4–6 and S4–S6), logicals to print residuals (printres = false) or *ɸ* at each time step (printphi = false), and the path name where *ɸ* will be output as a CSV (pathname = ‘cd’).

### Relaxed SAV algorithm and solver

For clarity, we retain the nomenclature for the relaxed SAV method of Jiang et al. [63] The nonlinear free energy:


$$\begin{array}{c} E(\phi )=\int_{\Omega }\left(\frac{\epsilon^{\text{2}}}{\text{2}}|\nabla \phi {|}^{\text{2}}+F(\phi )\right)dx \end{array}$$ (12)

Is recast with the following SAV over the spatial domain *Ω* at time *t*:


$$\begin{array}{c} (t)=\sqrt{\int_{\Omega }\left(F(\phi )-\frac{\gamma_{\text{0}}}{\text{2}}\phi^{\text{2}}\right)dx+C_{\text{0}}} \end{array}$$ (13)

where $\gamma_{\text{0}}\geq \;\text{0}$ and *C*_*0*_ ≥ 0 ensure that the square root in *r*(*t*) is well defined. $\gamma_{\text{0}}$ and *C*_*0*_ do not alter the underlying PDE bu*t* improve the unconditional energy stability of the numerical scheme. In the SAV code, we set *γ*_*0*_ = 2 and *C*_*0*_ = 1 as default values. Our empirical tests indicate these choices provide a good balance between accuracy and stability, which is consistent with previous results [35,63,69]. We follow the regularized SAV framework of Chen et al. [70] and its relaxed extension from Jiang et al. [63] by adding a quadratic term $\frac{\gamma_{\text{0}}}{\text{2}}\phi^{\text{2}}$ to the free energy E(ϕ), which is then subtracted after squaring the auxiliary variable *r*(*t*)*:*


$$\begin{array}{c} \tilde{E}(\phi ,r)=\int_{\Omega }\frac{\epsilon^{\text{2}}}{\text{2}}|\nabla \phi {|}^{\text{2}}dx+\frac{\gamma_{\text{0}}}{\text{2}}\phi^{\text{2}}+r^{\text{2}}-C_{\text{0}} \end{array}$$ (14)

The auxiliary variable *r*(*t*) in Equation 13 together with the modified energy $\tilde{E}$ in Equation 14 yield a linear system of equations with constant coefficients when using Crank-Nicolson time discretization to solve for *ɸ* and *r*:


$$\begin{array}{c} \frac{\phi^{n+\text{1}}-\phi^{n}}{\Delta t}=\Delta \mu^{n+\text{1}/\text{2}} \end{array}$$ (15)

$$\begin{array}{c} \mu^{n+\text{1}/\text{2}}=\gamma_{\text{0}}\phi^{n+\text{1}/\text{2}}-\Delta \phi^{n+\text{1}/\text{2}}+\frac{r_{*}^{n+\text{1}/\text{2}}}{Q\left(\phi_{*}^{n+\text{1}}\right)}V\left(\phi_{*}^{n+\text{1}}\right) \end{array}$$ (16)

$$\begin{array}{c} \frac{r_{*}^{n+\text{1}}-r^{n}}{\Delta t}=\int_{\Omega }\frac{V\left(\phi_{*}^{n+\text{1}}\right)}{\text{2}Q\left(\phi_{*}^{n+\text{1}}\right)}\frac{\phi^{n+\text{1}}-\phi^{n}}{\Delta t}dx \end{array}$$ (17)

where $\phi^{n+\text{1}/\text{2}}=\frac{\text{1}}{\text{2}}\left(\phi^{n}+\phi^{n+\text{1}}\right)$, $r_{*}^{n+\text{1}/\text{2}}=\frac{\text{1}}{\text{2}}\left(r^{n}+r_{*}^{n+\text{1}}\right)$, $\phi_{*}^{n+\text{1}}=\frac{\text{3}}{\text{2}}\phi^{n}-\frac{\text{1}}{\text{2}}\phi^{n-\text{1}}$, $V\left(\phi_{*}^{n+\text{1}}\right)=F^{\prime }\left(\phi_{*}^{n+\text{1}}\right)-\gamma_{\text{0}}\phi_{*}^{n+\text{1}}$, and$Q(\phi )=\sqrt{\int_{\Omega }\left(F(\phi )-\frac{\gamma_{\text{0}}}{\text{2}}\phi^{\text{2}}\right)dx+C_{\text{0}}}$. The terms $\phi_{*}^{n+\text{1}}$ and $r_{*}^{n+\text{1}}$ refer to provisional solutions for the next time step: *ɸ*^*n*+1^ and*r*^*n*+1^. The algorithm then applies a relaxation step to the provisional $r_{*}^{n+\text{1}}$:


$$\begin{array}{c} r^{n+\text{1}}=\xi r_{*}^{n+\text{1}}+(\text{1}-\xi )Q\left(\phi_{*}^{n+\text{1}}\right) \end{array}$$ (18)

where ξ ∈ [0, 1] is the real root of the quadratic:


$$\begin{array}{c} a\xi^{\text{2}}+b\xi +c=\text{0} \end{array}$$ (19)

with:


$$\begin{array}{c} a={\left(r_{*}^{n+\text{1}}-Q(\phi^{n+\text{1}})\right)}^{\text{2}} \end{array}$$ (20)

$$\begin{array}{c} b=\text{2}\left(r_{*}^{n+\text{1}}-Q(\phi^{n+\text{1}})\right)Q(\phi^{n+\text{1}}) \end{array}$$ (21)

$$\begin{array}{c} ={\left[Q(\phi^{n+\text{1}})\right]}^{\text{2}}-{\left(r_{*}^{n+\text{1}}\right)}^{\text{2}}-\eta \Delta t\int_{\Omega }\left[\mu^{n+\text{1}/\text{2}}\left(-\Delta \mu^{n+\text{1}/\text{2}}\right)\right]dx \end{array}$$ (22)

The term *η* ∈ [0, 1] scales the dissipation term from no dissipation (*η* = 0) to maximum dissipation (*η* = 1). In the SAV code, we take *η* = 0.95 as a default value, which was found to result in good accuracy and numerical stability [63]. With Equations 18–22, ξ is computed to minimize $\left|r^{n+\text{1}}-Q(\phi^{n+\text{1}})\right|$ by solving the following optimization problem:


$$\begin{array}{c} \xi_{\text{0}}={min}_{\xi \in [\text{0},\text{1}]}\xi ,s.t.\text{\textasciitilde{}}{{(r}^{n+\text{1}})}^{\text{2}}-{{(r}_{*}^{n+\text{1}})}^{\text{2}}\leq \eta \Delta t\;\int_{\Omega }\left[\mu^{n+\text{1}/\text{2}}(-\Delta \mu^{n+\text{1}/\text{2}})\right]dx \end{array}$$ (23)

Once $r^{n+\text{1}}$ is obtained through the above relaxation, *ɸ*^*n*+1^ is updated by Equation 15.

The SAV algorithm was coded to share global functions with the NMG solver as much as possible. ‘CahnHilliard_SAV’ takes an initial chemical state field (*ɸ*_*0*_; Box 1) provided by the user or created by ‘ch_initialization’ and uses ‘sav_solver’ [together with numpy.fft.fft2 (Python), fft2 (MATLAB), or fft (Julia FFTW) and other auxiliary functions in the package] to solve for *ɸ* at each time step *dt*. SAV solutions by FFT are intrinsically periodic. For Neumann boundary conditions, the domain is reflected once in each dimension (Fig 2C, inset), and a cosine boundary condition is enforced on the fourfold expanded domain to ensure zero flux. ‘CahnHilliard_SAV’ handles the following simulation parameters with the variable name and default value in parentheses: number of time iterations (t_iter = 1e3), the size of the time step (dt = 2.5e-5), the number of mesh points *m* for *ϵ*_*m*_ (m = 8), the boundary conditions (boundary = ‘periodic’), the domain size in terms of right and left coordinates of x and y (domain = [1 0 1 0]), logicals to print *ɸ* at each time step (printphi = false), the path name where *ɸ* will be output as a CSV (pathname = ‘cd’), the regularization parameter *C*_*0*_ (C0 = 1), the stabilization parameter *γ*_*0*_ (gamma0 = 2), the relaxation parameter *η* (eta = 0.95), and a logical for invoking relaxation (xi_flag = true).

### Finite difference solver

Outside of the Python, MATLAB, and Julia packages, we encoded a separate (not-recommended) set of functions in MATLAB that attempts to solve the Cahn-Hilliard equation by the forward Euler method of finite difference. ‘CahnHilliard_FD’ takes an initial chemical state field (*ɸ*_*0*_; Box 1) provided by the user or created by ‘ch_initialization’ and uses ‘fd_solver’ to estimate *ɸ*(*t* + *dt*) by linear extrapolation from *ɸ*(*t*). ‘CahnHilliard_FD’ handles the following simulation parameters with the variable name and default value in parentheses: number of time iterations (t_iter = 1e3), the size of the time step (dt = 2.5e-5), the spacing of timesteps that are printed or saved (dt_out = 10), the number of mesh points *m* for *ϵ*_*m*_ (m = 8), the boundary conditions (boundary = ‘periodic’), the number of smoothing relaxations done at the start of the simulation (c_relax = 2) and a logical variable to determine if the initial conditions are smoothed (presmooth = false), the domain size in terms of right and left coordinates of *x* and *y* (domain = [1 0 1 0]), logicals to print residuals (printres = false) or *ɸ* at each time step (printphi = false), and the path name where *ɸ* will be output as a CSV (pathname = ‘cd’).

### Spinodal decomposition

Square meshes of size 2^6^, 2^7^, 2^8^, or 2^9^ were randomly initialized with +1 at a probability of 25%, 50%, or 75% in Julia by randomizing +1 and –1 using the shuffle! function (random seed = 1234). The ‘relax’ function in the Julia package was then used to smooth the initial conditions with the following parameters: mu = zeros(GridSize,GridSize), dt = 6.25e-6, n_relax = 4, Lx = Ly = 1, m = 8, and boundary = “neumann”. These new smoothened initial conditions were used as *ɸ*_0_ for simulations. NMG used default values for tolerance and solver_iter. SAV used default values for *C*_*0*_, *γ*_*0*_, *η*, and regularization.

### Critical droplet simulations

Critical droplets were initialized on 2^7^ or 2^8^ square meshes with the ‘initialization’ function in the Julia package and method = ‘droplet’, which places a + 1 circle with radius *R*_*0*_ in the center of the domain surrounded by –1 and the equilibrium interface $\phi (R)=tanh\left(\frac{R_{\text{0}}-R}{\sqrt{\text{2}}\epsilon_{m}}\right)$ (Box 2). *ϵ*_*m*_ was altered by varying *m* = 4–48 and changing the mesh size (Eq 8). Simulations were performed with the ‘CahnHilliard_NMG’ function in the Julia package and tol = 1e-6, dt = 2.5e-5, solver_iter = 1e4 for 10 *t*_*char*_. Initial guesses of *R*_*0*_ were refined to three significan*t* digits upon identifying the threshold of critical droplet behavior for a given *ϵ*_*m*_. Consistency was confirmed by i) repeating a subset of simulations with the ‘CahnHilliard_SAV’ function in the MATLAB package and ii) altering the position of the droplet (S3 Fig, Panels A–B) with boundary = ‘periodic.’

To calculate *R*_*i*_ and *R*_*eq*_, *R*(*t*) was defined at every 10 time steps with contourc.m and levels = 0. *R*_*i*_ was interpolated as the *R*_*0*_ halfway between the largest R_0_ that dissipated and the smallest *R*_*0*_ that persisted for a given *ϵ*_*m*_. *R*_*eq*_ was gleaned from the inflection point of *R*(*t*) for droplets that still dissipated at a given *ϵ*_*m*_. We smoothed numerical fluctuations in *R*(*t*) by fitting a fifth-order polynomial to the transition zone of interest and calculating zeros of the second derivative analytically. Note that *R*_*i*_, *R*_*eq*_, and *ϵ*_*m*_ are defined relative to *L*_*X*_ = *L*_*Y*_ = 1. For other domains, *R*_*i*_, *R*_*eq*_, and *ϵ*_*m*_ should be multiplied by *L*, and to maintain the same time scale *D* should be multiplied by *L*^2^.

*R*_*eq*_ was regressed against *ϵ*_*m*_ with scipy.optimize.curve_fit using linear, hyperbolic, logarithmic, power-law, and hyperbolic-to-linear relationships. Alternative models were compared to the hyperbolic-to-linear fit by Bayes information criterion weights [71]. For the comparison between power-law and hyperbolic-to-linear relationships, *R*_*eq*_ vs. *ϵ*_*m*_ data were bootstrapped 1000 times to estimate 90% confidence intervals.

### Cell lines

HeLa cells (female) were cultured in Eagle’s Minimum Essential Medium (ATCC, CCL-2) supplemented with 10% fetal bovine serum (HyClone, SH303396.03). MCF10A-5E cells (female) [55] were grown in Dulbecco’s modified Eagle’s medium/F-12 (Gibco, 11330–032) supplemented with 100 ng/ml cholera toxin (Sigma, C8052), 20 ng/ml epidermal growth factor (Peprotech, AF-10015), 10 µg/ml insulin (Sigma, I1882), 500 ng/ml hydrocortisone (Sigma, H0888) and 5% horse serum (Gibco, 16050). All base media were supplemented with 1% penicillin/streptomycin (Gibco, 15140) and all cultures were grown with 5% CO_2_ in a humidified incubator at 37ºC. By STR profiling (ATCC), HeLa cells were authenticated to be derived from RRID:CVCL_0030 (93% match), and MCF10A-5E cells were authenticated to be derived from RRID:CVCL_0598 (100% match).

### Immunofluorescence of chromosome spreads after G2 release

10-cm dishes of cells at 70% confluency were treated with 2 mM thymidine (Sigma, T1895) in fresh medium for 24 hours. Cells were washed three times with 5 ml PBS, and 9 µM RO-3306 (Selleckchem, S7747) was added with fresh media for an additional 24 hours. For HeLa, cells were again washed three times with 5 ml PBS and trypsinized. For MCF10A-5E, cells were trypsinized in the presence of 9 µM RO-3306, and pelleted cells were washed three times with 5 ml PBS. Pelleted cells were then transferred to fresh medium and incubated at 37°C for 10 minutes (HeLa) or 70 minutes (MCF10A-5E). Cells were again pelleted and then hypotonically swelled in 75 mM KCl, 0.8% Na Citrate, and H_2_O in a 1:1:1 ratio for 15 minutes at room temperature. Swollen cells were centrifuged onto coverslips at 67 rcf for 5 minutes with a Cytospin 4 (Thermo Shandon) and transferred to 6-well plates for staining. Samples were fixed with 2% paraformaldehyde (Thermo Fisher, 50980489) for 20 minutes, blocked with 1% (w/v) bovine serum albumin (Fisher BioReagents) in PBS for 1 hour at room temperature, and stained for AURKB (BD Biosciences, RRID:AB_398396; 1:250 dilution), Centromere Antigen (Antibodies Incorporated, RRID:AB_2939058; 1:500 dilution), and phospho-Histone H3 (Thr3) (Abcam, RRID:AB_1566301; 1:250 dilution) in PBS + 0.1% Tween-20 (Sigma) (PBS-T) overnight at 4°C. Samples were washed three times with PBS-T for 5 minutes and then incubated for 1 hour at room temperature with the following secondary antibodies diluted in PBS-T: Alexa Fluor 488-conjugated goat anti-mouse (Thermo Fisher, RRID:AB_2534088; 1:1000 dilution), Alexa Fluor 568-conjugated goat anti-human (Thermo Fisher, RRID:AB_2535746; 1:1000 dilution), and Alexa Fluor 647-conjugated goat anti-rabbit (Thermo Fisher, RRID:AB_2535813; 1:1000 dilution). Samples were washed two times with PBS-T for 5 minutes, counterstained with 1 µg/ml DAPI (Thermo Fisher, 62248; 1:1000 dilution) for 10 minutes, washed two times with PBS-T, and mounted on glass slides with ProLong Gold antifade reagent (Invitrogen, P36934) for imaging.

Mounted coverslips were imaged as 7–11 optical sections on a Leica STELLARIS 5 LIAchroic confocal laser-scanning microscope with a 63x 1.4 NA plan apochromat oil-immersion objective and the following acquisition parameters: 60.13 nm pixel size at 3x optical zoom; 1000 x 1000 pixels^2^ field of view; 475 ns pixel dwell time; 330 nm z step size; 1 Airy Unit pinhole for a 520 nm emission; line accuracy = 2; 405 nm laser power = 1.7%; 488 nm laser power = 10%; 561 nm laser power = 5%; 638 nm laser power = 0.1–1%; and all detectors in photon-counting mode. Image stacks were deconvolved with Leica LIGHTNING deconvolution software using default parameters and Prolong Gold as the immersion medium.

To quantify CPC immunoreactive foci, optical sections that transected spread chromosomes were maximum-intensity projected, and AURKB foci between sister chromatids were selected with the magic wand tool in ImageJ (tolerance = 40 [HeLa] and 25 with 8-connected mode [MCF10A-5E]). Segmented areas were converted to radii of equivalent circles for downstream analysis. Line scans were performed with the freehand line tool in ImageJ (width = 5 pixels), and peaks in the exported traces were identified with the Python function ‘scipy.signal.find_peaks’ using intensity values rescaled by max intensity with prominence = 15 (rescaled by max intensity), min_width = 100 nm, and max_width = 800 nm.100 bootstraps of 50% subsampled images (*N =* 10 for Hela and *N* = 38 for MCF10A-5E) were generated using numpy.random.choice (Python) without replacement. Half of the subsampled images were used to constrain *ϵ*_*m*_ from *R*_*eq*_ (Figs 4D and S6, Panel B), and the remaining half were used for line scan analysis (Figs 6E, 6H and S6, Panel D). Using the bootstrapped distributions of drop-to-drop distances from experiments, we binned the mean drop-to-drop distance as a histogram and estimated a 95% confidence interval. The optimal bin width was determined by comparing the 95% intervals to the average Gaussian kernel density estimate (using scipy.gaussian_kde) from these experimental bootstraps, which gave ~55 bins over the range 0–3.5 µm.

### CPC droplet simulations

CPC simulations were initialized with a +1-state vertical stripe of width *W* at the midpoint overlaid with a +1-state circle with radius *R*_*IC*_ in the center of the domain (*L*_*x*_ = *L*_*y*_ = 2 = 6.4µm, GridSize = 512). The rest of the domain was filled with the –1 state. Reasonable values of *R*_*IC*_ were selected from HeLa measurements of the inner centromere (10 values between 50–350nm), and *W* values ranging from 50–140 nm. Irregular width simulations were initialized with the same values for *R*_*IC*_; *W* was selected from a normal distribution (*µ* = 90 nm, *σ* = 10 nm) at each vertical position and initialized for five simulations with the random seed set to the simulation number (50 total). Visualizations in Figs 5 and 6A–6B show the central 3.2 µm of the domain—lateral mesh points outside this range factor into the calculations but do not change from their initial conditions. All simulations were run with *dt* = 1.53e-6 for 0.04 or 0.4 *t*_*char*_ (~7 or 70 minutes), which heuristically gave stable and accurate simulations for the resolution *dx* = 1/512 as determined by comparisons between SAV and NMG. For NMG, tolerance = 1e-5 and solver_iter = 1e4.

To calculate drop-to-drop separation for each irregular-width simulation, we identified the center of droplets by calculating the mean location of each contour with contourc.m (level = 0) and tracked each droplet throughout the simulation. Time points within each simulation were randomly sampled (without replacement) so that the number of timepoints multiplied by the number of simulations approximated the number of chromosomes for each cell line. Each time point was then randomly paired with a chromosome arm length measured in the cell line of interest (S4 Fig, Panel A), and the distances between droplet centers were calculated for any droplets vertically within the paired arm-length distance from the inner centromere.

### Phos-Tag immunoblotting

Cells were synchronized with thymidine then RO-3306 and released for 10 minutes (HeLa) or 70 minutes (MCF10A-5E) as described above. Cells were counted using an automated cell counter (Invitrogen), lysed with Laemmli sample buffer (62.5 mM Tris-HCl (pH 6.8), 2% SDS, 10% glycerol, 100 mM dithiothreitol, and 0.01% bromophenol blue), and sheared with a 25-gauge needle (BD PrecisionGlide). Phos-tag immunoblotting of 50,000 cells per lane was performed on polyacrylamide gels of 6% (INCENP), 8% (AURKB and CDCA8), or 15% (BIRC5) containing 10 mM Phos-tag acrylamide (Nard Institute, AAL-107) and 0.1 mM MnO_4_•4H_2_O. Gels were run with WIDE-VIEW prestained protein marker (FUJIFILM Wako, 230–02461) under 40 mA constant current for 70–90 minutes. Before electrophoretic transfer, the gels were incubated in transfer buffer (25 mM Tris, 192 mM glycine, 0.0375% SDS) plus 1 mM EDTA for 10 minutes, followed by an additional 10-minute incubation in transfer buffer. Proteins were tank transferred to a PVDF membrane (Millipore) in transfer buffer plus 10% methanol (INCENP, AURKB, and CDCA8) or 20% methanol (BIRC5) at 100 V for 60 minutes on ice. Membrane blocking and antibody dilution were performed with 5% low-fat milk powder in Tris-buffered saline (TBS) containing 0.1% Tween 20 solution (TBS-T). Immunoblotting and secondary fluorescence or chemiluminescence detection was completed as described [72] with primary antibodies for AURKB (BD Biosciences, RRID:AB_398396; 1:1000 dilution), BIRC5 (Cell Signaling, RRID:AB_2063948; 1:1000 dilution), CDCA8 (P.T.S. custom rabbit polyclonal [73]; 1:2000 dilution), GAPDH (Ambion, RRID:AB_437392; 1:10,000 dilution), INCENP (Cell Signaling, RRID:AB_2127513; 1:1000 dilution), and vinculin (Millipore, RRID:AB_11212640; 1:10,000 dilution). Immunoblots were quantified in ImageJ as previously described [72]. The intensity minimum between peaks was used to separate the upper and lower forms of CDCA8 and INCENP. Original uncropped blots are included in S1 raw gel.

### Data, materials, and software availability

Code used to generate the results and figures in this paper is available on GitHub (https://github.com/JanesLab/GrovesSM_CahnHilliard). Python, Julia, and MATLAB packages for modeling the Cahn-Hilliard equation, complete with tutorials, are available on GitHub (https://github.com/JanesLab/CahnHilliard_NMGSAV). Source data for this work are available on LabArchives (http://dx.doi.org/10.25833/a7vd-t477).

## Supporting information

S1 Fig Extended spinodal decompositions comparing different solvers. (A, B) Time evolution for the relative energy [*E*(*t*) / *E*(*t* = 0)] (A) and mass error [*M*(*t*) – *M*(*t* = 0)] (B) of the spinodal decompositions in Fig 1. (C) Time evolution for the same spinodal decomposition as in Fig 1 but with an implicit finite difference solver. (D) Runtime performance for the implicit finite difference solver compared to NMG. (E, F) Time evolution for the same spinodal decomposition as in Fig 1 but with periodic boundary conditions. (G, H) Time evolution for the relative energy (E) and mass error (F) of the spinodal decompositions in (C, D). (I) A 50:50 initialization of ±1 chemical states with zero (left), two (center), or four (right) rounds of smoothing. (J) Energy minimizations of spinodal decomposition are consistent and stable after at-least one round of smoothing. Root mean squared error (RMSE) is shown as a function of the number of smoothing operations. The level of smoothing used in the indicated figures is highlighted.
(EPS)

S2 Fig Alternative initial conditions for spinodal decomposition, NMG–SAV error, and performance for different mesh sizes. (A, B) Snapshots of spinodal decomposition for initial conditions with 75% + 1 state (A) or 25% + 1 state (B) and periodic (left) or Neumann (right) boundary conditions. (C, D) Runtime performance across mesh sizes (2^6^–2^9^) for NMG (C) and SAV (D) solvers in Python, MATLAB, and Julia for spinodal decompositions initialized with *N* = 3 random mixtures of ±1 chemical states (25%, 50%, and 75% condensed phase) and either periodic or Neumann boundary conditions. All simulations were performed on AMD EPYC 9454 processors (3.81 GHz max, 48 cores per socket, 2 sockets) with up to 300 (Python and MATLAB) or 1000 (Julia) GB RAM allocated, using up to 16 cores for computation on a 2^7^ x 2^7^ mesh (*L*_*X*_ = *L*_*Y*_ = 1) for 2000 time steps (*dt* = 5.5e-6) and *ϵ*_*m*=8_. (E, F) Root mean squared error (RMSE) between NMG and SAV solutions for different mesh sizes (2^6^–2^8^) and a large mesh (2^9^, inset) when *dt* = 5.5e-6 (solid) and *dt* = 5.5e-7 (dashed). Note for the large mesh that RMSE decreases considerably with the finer time step for 25% (green) and 50% (orange) condensed phase. (G, H) Power law relationship between the characteristic length scale (*L*_*char*_, estimated by FFT) and *t*_*char*_ for the 2^9^ x 2^9^ mesh results shown in Panels C–D. *R*^2^ goodness of fit was greater than 0.9 for both solvers and both boundary conditions.
(EPS)

S3 Fig Robustness of critical radii and fit of *R*_*eq*_ compared to alternatives. (A, B) Identical simulations as in Fig 3B but with an off-center droplet (A) or a droplet split across a periodic boundary (B). *R*_*0*_ = 0.105 is shown as an example. (C–F) The hyperbolic-to-linear (H2L) fit of the data in Fig 3E versus a linear (C), logarithmic (D), hyperbolic (E), or power-law (F) model. Fits were compared by Bayes information criterion (BIC), and BIC weights [71] (BICw) are shown indicating the relative likelihood of each alternative compared to H2L. In (F), models are compared with bootstrapped data from Fig 3E, and BICw is reported with 90% confidence intervals (CI) estimated from 1000 bootstrap replications.
(EPS)

S4 Fig Mapping the Cahn-Hilliard equation to physical dimensions of (pro)metaphase chromosomes and the CPC. (A) Size distribution of spread chromosomes prepared from HeLa cells and MCF10A-5E cells. Data are from *N* = 231 chromosomes from 10 images for HeLa and *N* = 218 chromosomes from 38 images for MCF10A-5E. Distributions were compared by KS test. (B) Plausibility of the alpha-helical “dogleash” subunit of the CPC spanning condensed and soluble phases. Given a 3.2 µm arm length (A) x 2 arms = 6.4 µm domain, *ϵ*_*m*_ = 21.6 ± 0.6 nm for HeLa and *ϵ*_*m*_ = 28.5 nm ± 1.1 nm for MCF10A-5E (Eq 8).
(EPS)

S5 Fig Estimation and perturbation of the pH3T3 width. (A) Putative configuration of the cohesin complex with H3T3 kinase. The cohesin ring holds together sister chromatids at different orientations, and one of its regulatory subunits binds the unstructured N terminus of H3T3 kinase [49,50]. Length estimates are from [48,51]. (B) Kinetochore perturbation described by Gascoigne et al. [52]. HeLa cells transiently coexpressing GFP-CENP-C-∆C-H2B and mCherry-CENP-T-∆C-H2B were compared to control cells transiently coexpressing GFP-H2B and mCherry-H2B. (C, D) Enlarged and reprocessed images from S3C Fig of [52]. Reprinted with permission from Cell Press.
(EPS)

S6 Fig HeLa- and MCF10A-5E-specific differences in CPC properties. (A) Histogram of non-IC foci (gray) and IC foci (white and orange) quantified by idealized circular radius from *N* = 218 chromosomes in 50 MCF10A cells. The 95th percentile of the non-IC droplet size distribution defining *R*_*eq*_ is shown. The distribution of non-IC foci (*N* = 1223) and IC foci (*N* = 268) were compared by KS test. (B) Estimation of *ϵ*_*m*_ from measured *R*_*eq*_ using the hyperbolic-to-linear regression (Fig 3E) scaled for a 3200-nm physical spatial domain. The interquartile range (IQR) was calculated by 50% subsampling of *N* = 38 MCF10A-5E cells for 100 iterations without replacement and propagating to the *ϵ*_*m*_ estimate. (C) Cumulative density function (CDF) plot of drop-to-drop distances from Cahn-Hilliard simulations with *ϵ*_*m*_ = 21.6 nm (dotted line, *N* = 1221 distances) or 28.5 nm (solid line, *N* = 839 distances). The distributions were compared by KS test. (D) Median CDF plot ± 95% bootstrapped confidence interval (blue) of peak-to-peak separation for CPC foci in HeLa cells (dotted line, *N* = 583 peaks) or MCF10A cells (solid line, *N* = 831 peaks). The distributions were compared by KS test. (E) Representative Phos-Tag immunoblots of whole-cell extracts (50,000 cells) from HeLa and MCF10A-5E cells. Lysates were immunoblotted for the indicated targets with GAPDH or vinculin used as loading controls. (F) Quantitative densitometry of (E) for *N* = 5 biological replicates of each cell line. *P < 0.05, **P < 0.01, ***P < 0.0001 by unpaired two-sample *t* test.
(EPS)

S1 Movie Droplet dynamics for the indicated initial radii (*R0*) and interfacial energy coefficient (*ε*) over the indicated characteristic time (*t*). (MP4)

S2 Movie CPC dynamics initialized with a rectangular crosshair over the indicated time (*t*). (MP4)

S3 Movie CPC dynamics initialized with a uniform or an irregular width (*W*) over the indicated time (*t*). (MP4)

S1 Raw Gel Uncropped immunoblot images. (PDF)
