# Supplementary material for: Cell-specific Cahn-Hilliard models predict condensed fates of the chromosomal passenger complex
Source: PLoS Comput Biol. 2026 Aug 3;22(8):e1014568. doi: 10.1371/journal.pcbi.1014568 (PMC13446744; doi:10.1371/journal.pcbi.1014568)
Supplement: Supplementary file 1 [file pcbi.1014568.s010.pdf]

Fig. S6E

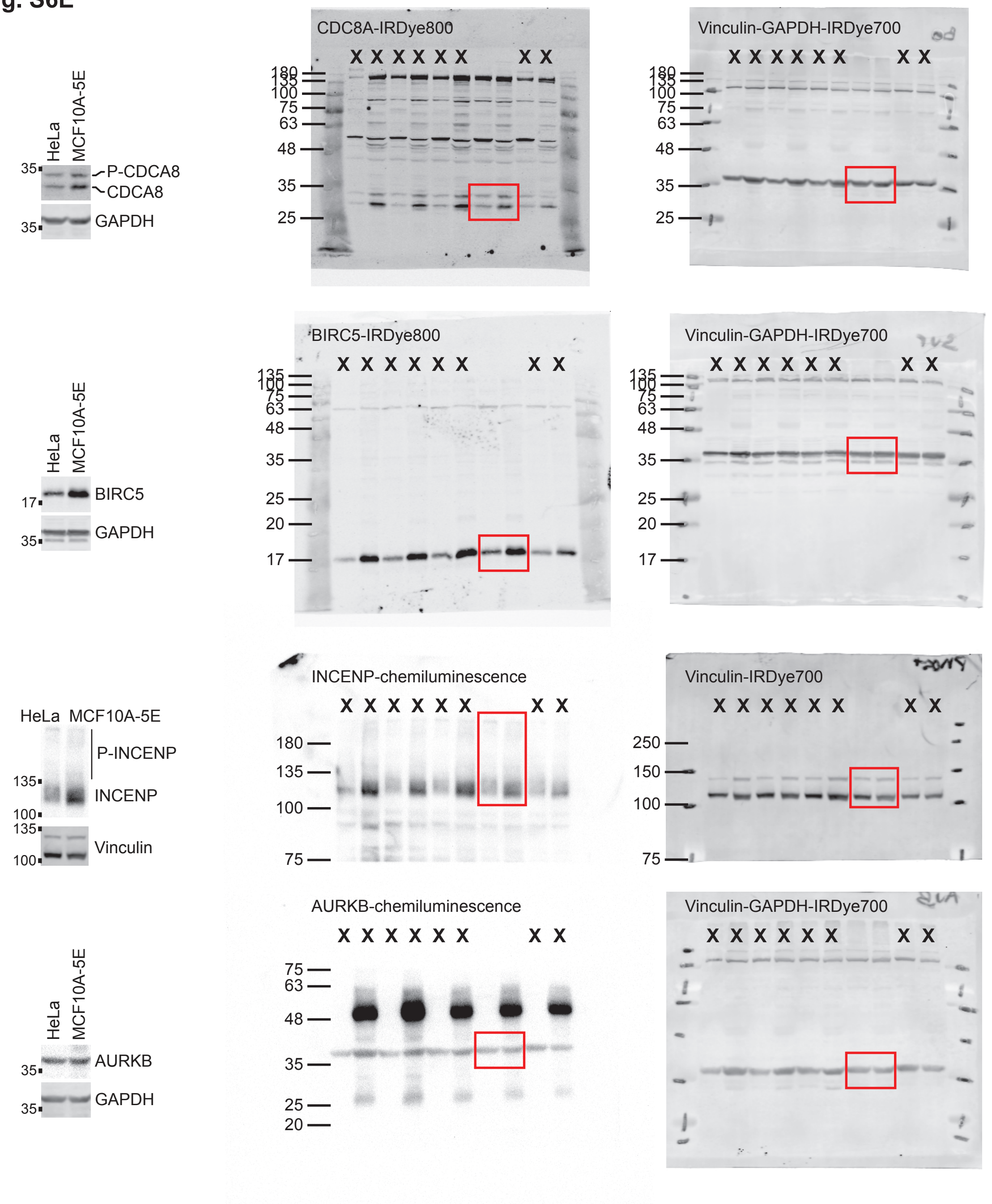
HeLa
MCF10A-5E
P-CDCA8
CDCA8
GAPDH
BIRC5
P-INCENP
INCENP
Vinculin
AURKB
CDC8A-IRDye800
Vinculin-GAPDH-IRDye700
BIRC5-IRDye800
INCENP-chemiluminescence
Vinculin-IRDye700
AURKB-chemiluminescence
180
135
100
75
63
48
35
25
20
17
250
150
