# Supplementary material for: Cell-specific Cahn-Hilliard models predict condensed fates of the chromosomal passenger complex
Source: PLoS Comput Biol. 2026 Aug 3;22(8):e1014568. doi: 10.1371/journal.pcbi.1014568 (PMC13446744; doi:10.1371/journal.pcbi.1014568)
Supplement: Supplementary file 2 [file pcbi.1014568.s011.pdf]

MANUSCRIPT ID: PCOMPBIOL-D-25-02313

TITLE: Cell-specific Cahn-Hilliard models predict condensed fates of the chromosomal passenger complex

AUTHOR(S): Sarah M. Groves, Min-Jhe Lu, Astrid Catalina Alvarez-Yela, P. Todd Stukenberg, John S. Lowengrub, Kevin A. Janes

The manuscript under review introduces fast, stable Cahn–Hilliard solvers (NMG and SAV) in Python, MATLAB, and Julia to model biomolecular phase separation. Using extensive simulations, the authors derive a general relationship between the interfacial energy coefficient and critical droplet size. Applying this to the chromosomal passenger complex (CPC), they use measured CPC droplet sizes to fully parameterize the model and show that CPC dynamics during mitosis, breakup, coarsening, and final centromere localization, are accurately predicted by simple Cahn–Hilliard physics. This establishes CPC as a phase-separated condensate and provides a general computational framework for studying similar systems.

The manuscript is clearly written, and the authors introduce the model in a structured and accessible manner before connecting it to CPC condensate dynamics. However, I have several concerns outlined below that should be addressed before I can recommend the manuscript for publication.

1> The manuscript introduces the Flory–Huggins free-energy formulation, then moves to a double-well potential, and finally presents the Cahn–Hilliard equation. This progression is interesting, but the motivation for using the Flory–Huggins form instead of beginning directly from a Landau–Ginzburg free-energy functional is not entirely clear to me. Could the authors elaborate on why this route was chosen, and what advantages it offers for this particular biological system?

2> In your simulations of the Cahn–Hilliard dynamics starting from a random initial distribution, the domain size $l(t)$ is expected to grow as $l(t) \sim t^{1/3}$ according to the classical Lifshitz–Slyozov theory. It would be very helpful if you could show the time evolution of the characteristic length scale, e.g., extracted from the structure factor peak or real-space correlation function—to confirm the expected $t^{1/3}$ scaling. Could you please comment on whether your numerical scheme reproduces this coarsening law ?
Ref: **A. J. Bray**, *"Theory of Phase-Ordering Kinetics"*, **Advances in Physics**, 43, 357–459 (1994).

3> Could the authors clarify how the spectral (Fourier) method is applied on a non-periodic domain? The manuscript mentions a reflection-based approach, but the implementation is not fully clear, especially regarding how the non-periodic boundary conditions are enforced.

4> In Figure 3(D and E), the power-law behavior is shown on a linear plot. Presenting the same data in a log–log plot could make the scaling more visually clear and quantitatively verifiable.

5> Could the CPC condensate (droplet) size be connected to Rayleigh Plateau Instability? Can the authors connect it?

6> The manuscript introduces width irregularities in the simulations, but it is unclear whether these perturbations are imposed uniformly or derived from experimentally measured variations.

7> The manuscript does not discuss how the simulation outcomes depend on system size since both the R_i and the ϵ_m (interface energy coefficient) can be significantly influenced by the size of the computational domain.
Ref: Yue P, Zhou C, Feng JJ. Spontaneous shrinkage of drops and mass conservation in phase-field simulations. J Comput Phys. 2007;223(1):1–9. doi: https://doi.org/10.1016/j.jcp.2006.11.020.

8> Should the take away message be that the separation between the droplet is non-uniform and it will originate from the non-uniform width of the CPC?
