# Supplementary material for: Cell-specific Cahn-Hilliard models predict condensed fates of the chromosomal passenger complex
Source: PLoS Comput Biol. 2026 Aug 3;22(8):e1014568. doi: 10.1371/journal.pcbi.1014568 (PMC13446744; doi:10.1371/journal.pcbi.1014568)
Supplement: Supplementary file 3 [file pcbi.1014568.s012.docx]

**RESPONSE TO REVIEWERS**

Reviewer #1

*The manuscript under review introduces fast, stable Cahn–Hilliard solvers (NMG and SAV) in Python, MATLAB, and Julia to model biomolecular phase separation. Using extensive simulations, the authors derive a general relationship between the interfacial energy coefficient and critical droplet size. Applying this to the chromosomal passenger complex (CPC), they use measured CPC droplet sizes to fully parameterize the model and show that CPC dynamics during mitosis, breakup, coarsening, and final centromere localization, are accurately predicted by simple Cahn–Hilliard physics. This establishes CPC as a phase-separated condensate and provides a general computational framework for studying similar systems. The manuscript is clearly written, and the authors introduce the model in a structured and accessible manner before connecting it to CPC condensate dynamics. However, I have several concerns outlined below that should be addressed before I can recommend the manuscript for publication.*

We thank Reviewer #1 for their provisional enthusiasm.

*1> The manuscript introduces the Flory–Huggins free-energy formulation, then moves to a double-well potential, and finally presents the Cahn–Hilliard equation. This progression is interesting, but the motivation for using the Flory–Huggins form instead of beginning directly from a Landau–Ginzburg free-energy functional is not entirely clear to me. Could the authors elaborate on why this route was chosen, and what advantages it offers for this particular biological system?*

An aim of this manuscript was to reach quantitative biochemists and cell biologists by starting with basic polymer physics before introducing soluble/condensed states and then the notion of diffuse interfaces. The Landau–Ginzburg free-energy functional abstracts the latter two topics simultaneously, which we feared would be off-putting for the intended audience. Another advantage of Flory–Huggins is that it accommodates different mixing energies, which could give rise to asymmetric double-well potentials beyond the symmetric one used in the current work.

*2> In your simulations of the Cahn–Hilliard dynamics starting from a random initial distribution, the domain size l(t) is expected to grow as l(t) ∼ t^1/3^ according to the classical Lifshitz–Slyozov theory. It would be very helpful if you could show the time evolution of the characteristic length scale, e.g., extracted from the structure factor peak or real-space correlation function—to confirm the expected t^1/3^ scaling. Could you please comment on whether your numerical scheme reproduces this coarsening law? Ref: A. J. Bray, “Theory of Phase-Ordering Kinetics”, Advances in Physics, 43, 357–459 (1994).*

To investigate whether the simulated spinodal decompositions were consistent with Lifshitz–Slyozov theory, we calculated the characteristic length scale for Julia NMG and SAV simulations on a 2^9^ x 2^9^ mesh (*L_X_* = *L_Y_* = 1) for 2000 time steps (*dt* = 5.5e-6 *t_char_*) and periodic or Neumann boundary conditions for the 50-50 initializations from S2 Fig, Panels C–D. We computed the characteristic length scale (*L_char_*) from the structure factor peak (*k_max_*) as *L_char_* = 2π / *k_max_* and found that *L_char_* grows roughly with the cubic root of time (S2 Fig, Panels G–H of the revision). The power law dependence is approximate due to the mesh discretization of the spatial domain and the intractability of using meshes greater than 2^9^. We revised the text as follows:

These overall trends persisted for mesh sizes ranging from 2^6^ x 2^6^ to 2^9^ x 2^9^ (S2 Fig, Panels C–D), where we confirmed that NMG and SAV were internally consistent throughout (S2 Fig, Panels E–F). Using results from the largest mesh, we verified that the characteristic length scale of spinodal decomposition grows with *t_char_*^~1/3^ (S2 Fig, Panels G–H), consistent with Lifshitz-Slyozov theory [44]. Because of their high initial spatial frequencies, spinodal decompositions are among the most difficult Cahn-Hilliard solutions, making them useful to discern whether the time step is appropriate for a given mesh size.

*3> Could the authors clarify how the spectral (Fourier) method is applied on a non-periodic domain? The manuscript mentions a reflection-based approach, but the implementation is not fully clear, especially regarding how the non-periodic boundary conditions are enforced.*

Thank you for bringing up this oversight in the Materials and Methods. We have revised the “Relaxed SAV algorithm and solver” section as follows:

The SAV algorithm was coded to share global functions with the NMG solver as much as possible. ‘CahnHilliard_SAV’ takes an initial chemical state field (*ɸ_0_*; Box 1) provided by the user or created by ‘ch_initialization’ and uses ‘sav_solver’ [together with numpy.fft.fft2 (Python), fft2 (MATLAB), or fft (Julia FFTW) and other auxiliary functions in the package] to solve for *ɸ* at each time step *dt*. SAV solutions by FFT are intrinsically periodic. For Neumann boundary conditions, the domain is reflected once in each dimension (Fig 2C, inset), and a cosine boundary condition is enforced on the fourfold expanded domain to ensure zero flux. ‘CahnHilliard_SAV’ handles the following simulation parameters with the variable name and default value in parentheses…

*4> In Figure 3(D and E ), the power-law behavior is shown on a linear plot. Presenting the same data in a log–log plot could make the scaling more visually clear and quantitatively verifiable.*

The log–log plot compresses the deviation between the theoretical *R_i_* vs. *ϵ_m_* in Fig. 3D, so we retained the linear display in the main figure. However, in the supplement, we agree that comparison of the H2L fit to others is better demonstrated on a log–log plot and have updated S3 Fig accordingly. We also appended a comparison to a power-law fit, which was equally good at relating *ϵ_m_* to *R_eq_* (bootstrapped BICw for power law: 90% CI = [2.9e-5,~1]; BICw for H2L: 90% CI = [6.9e-4, ~1]; S3 Fig, Panel F).

*5> Could the CPC condensate (droplet) size be connected to Rayleigh Plateau Instability? Can the authors connect it?*

We dwelled on this question considerably given that we had alluded to a connection in the original submission. First, the 2D Cahn-Hilliard simulations of Fig 5 do not model the Rayleigh-Plateau instability that arises in 3D because of circumferential surface tension. Instead, they capture spinodal dewetting caused by a thermodynamically favorable phase separation along the length of the column. Revisiting the data from Fig 5, we found that the spacing of droplets once formed does not depend on the CPC width *W* (not shown), contrasting the linear dependence predicted by theory for a Rayleigh-Plateau instability [1].

A separate question is whether the experimentally observed CPC droplets in Fig 4 and 6 are consistent with a Rayleigh-Plateau instability. FRAP measurements of CPC condensates [2] suggest a diffusivity about 100 times that of the cytoplasm. The Stokes–Einstein equation thus predicts a ratio of *µ*’/*µ* (viscosity of thread/viscosity of the medium) of 100, giving rise to a proportionality constant (*β*) between the droplet spacing (*λ*) and thread radius (*a*) of *β* = 12.93 x 2 ~ 26 based on the work of Tomotika [1]. By contrast, the median observed *λ* for the CPC is ~600 nm (Fig 6E,H) and the best estimate of *a* is 84 nm / 2 = 42 nm (S5 Fig, Panel A), yielding *β* = 600/42 ~ 14, which only occurs when *µ*’/*µ* = 5–10. The predicted droplet size (*R_drop_*) is related to *β* and *a* as follows: $R_{drop}=\left( \frac{3}{4}\beta\right)^{1/3}a$. *R_drop_* = 112 nm when *β* = 26, and *R_drop_* = 92 nm when *β* = 14; either value is within range of the transient droplets measured outside of the inner centromere (Fig 4C, top). Based on the larger discrepancy of *β* and the fact that *R_drop_* is not discriminatory given experimental uncertainty, we hesitate to make any strong assertions about whether the CPC undergoes a Rayleigh-Plateau instability and have removed this language from the revision.

*6> The manuscript introduces width irregularities in the simulations, but it is unclear whether these perturbations are imposed uniformly or derived from experimentally measured variations.*

Yes, we could have done a better job of explaining the rationale here. We have revised the following passage to make clear that irregularities come from the H3T3 kinase’s unstructured N terminus (S5 Fig, Panel A):

In the idealized case, transient droplets appear and disappear with perfect symmetry on either side of the inner centromere (Fig 6A). Biologically, however, there is uncertainty about the precise location of the H3T3 kinase (S5 Fig, Panel A), and pH3T3 marks are spatially rougher [59]. Therefore, we sampled *W* from a normal distribution (*µ* = 90 nm, *σ* = 10 nm) for each mesh row at its midpoint, which yielded an irregular pattern of longer-lived droplets (Fig 6B and S3 Movie).

*7> The manuscript does not discuss how the simulation outcomes depend on system size since both the 𝑅! and the 𝜖" (interface energy coefficient) can be significantly influenced by the size of the computational domain. Ref: Yue P, Zhou C, Feng JJ. Spontaneous shrinkage of drops and mass conservation in phase-field simulations. J Comput Phys. 2007;223(1):1–9. Doi:* [*https://doi.org/10.1016/j.jcp.2006.11.020*](https://doi.org/10.1016/j.jcp.2006.11.020)*.*

Yes, we were aware of this publication when performing the study. The computational domain defines the bulk into which the droplet may solubilize. It is why we defined *R_0_* relative to the domain size *L*. If *L* is not one, the Cahn-Hilliard dynamics are identical if the other terms in the equation are scaled appropriately. To illustrate, we reproduced Fig 3B with a domain size of *L* = [0,2]. If the grid size, *R_0_*_,_ and *ϵ_m_* are similarly doubled and mobility is increased fourfold to reflect diffusion in the x and y dimensions, the critical equilibrium radius simulations are identical:

**Fig R1.** Resimulation of Fig 3B for *L_X_* = *L_Y_* = 2. Mobility (*D*) was quadrupled and the normalized radius and ϵ_m_ are reported relative to the domain.

We modified the Critical droplet simulations section of the Materials and Methods as follows:

To calculate *R_i_* and *R_eq_*, *R*(*t*) was defined at every 10 time steps with contourc.m and levels = 0. *R_i_* was interpolated as the *R_0_* halfway between the largest R_0_ that dissipated and the smallest *R_0_* that persisted for a given *ϵ_m_*. *R_eq_* was gleaned from the inflection point of *R*(*t*) for droplets that still dissipated at a given *ϵ_m_*. We smoothed numerical fluctuations in *R*(*t*) by fitting a fifth-order polynomial to the transition zone of interest and calculating zeros of the second derivative analytically. Note that *R_i_*, *R_eq_*, and *ϵ_m_* are defined relative to *L_X_* = *L_Y_* = 1. For other domains, *R_i_*, *R_eq_*, and *ϵ_m_* should be multiplied by *L*, and to maintain the same time scale *D* should be multiplied by *L*^2^.

*8> Should the takeaway message be that the separation between the droplet is nonuniform and it will originate from the non-uniform width of the CPC?*

The physical concepts of dewetting and coarsening are entirely new to the CPC field, because phase separability of the CPC is still actively debated [2, 3]. To us, it is remarkable that biophysically constrained Cahn-Hilliard models capture real CPC data in absolute units (Fig 6E,H), which is why we framed those results as a key takeaway. We hesitate to read too much into droplet separation on its own, because biologically these short-lived condensates are removed from the key functions of the CPC at the inner centromere.

Reviewer #2

*In this work, the authors have built solver libraries for the Cahn-Hilliard equation for Python, MATLAB, and Julia using two different algorithms. The package was then used to explore the problem of condensation of the chromosomal passenger complex on mitotic chromosomes. The simulation results match experimental results. Although the methods here are not necessarily novel, I find the paper to be an interesting read and the open-source algorithms can be useful for other researchers, as phase-field models are becoming increasingly more used in the context of biomolecular separation processes. Below, I have some comments, requests for clarifications, and suggestions to the authors that I believe would improve the manuscript.*

We thank Reviewer #2 for their enthusiasm.

*>1 Although the title of the paper is related to the specific problem being studied (i.e., CPC condensates), the introduction barely mentions the problem in question or previous related results, some which are outlined later in page 19. The authors should expand the introduction portion of the manuscript to include the problem of CPC condensation, expanding the related bibliography review.*

Yes, we debated whether adding too much information about the CPC in the Introduction would detract from the methodological focus of the work, but the point is well taken about the title. We have expanded the Introduction as follows:

For most two-phase phenomena, there is not an exact solution for the Cahn-Hilliard equation. Numerical approximations are possible, but they require specialized approaches to address space/time-step restrictions, local nonlinearities, and nonconvexity. Published algorithms demonstrate proof of concept but historically do not include freely available code for redeployment in computational biology. Rare exceptions [18-20] are limited to specific boundary conditions that may not always be biologically appropriate. A general toolkit for Cahn-Hilliard models may facilitate its application to other examples of biological condensation [7], including settings where phase separation is actively debated.

One such example is the Chromosomal Passenger Complex (CPC), a heterotetramer that coordinates mitotic events by concentrating at the inner centromere during (pro)metaphase [21]. When recruited to chromatin above a critical concentration, the CPC has been suggested to form a biomolecular condensate that is critical for its inner centromeric functions [22]. However, cell biology techniques are limited in their ability to distinguish true condensates from other protein-concentrating interactions, and phase separability of the CPC was recently questioned [23]. One potentially discriminating test is to ask whether the CPC behaves as a Cahn-Hilliard fluid on mitotic chromatin.

Toward modeling the dynamics of biomolecular condensates within cells [24, 25], here we assemble and validate a multi-language suite [26] of numerical methods for solving the Cahn-Hilliard equation…

*>2 In page 10, the authors suggest that their CH package is more suitable for biology-focused users, without providing a reason for that claim. I was not able to identify biology-focused features in the solvers in question. The authors should elaborate more on that claim.*

The major deficiency we sought to get across was that packaged, redeployable functions are lacking in programming languages used by computational biologists. We revised the text as follows:

Various custom methods solve the Cahn-Hilliard equation accurately and efficiently through different numerical schemes (reviewed in [36]), but none are available with biology-focused users in mind. Current solvers are derived mathematically and described in pseudocode or illustrative scripts rather than functions packaged for general use. We thus developed generalized code for one finite-difference method and one spectral method in three widely used programming languages for computational biology: Python [37], MATLAB, and Julia [38].

*>3 In Figure 1, the authors determine the time step to guarantee stable diffusion dt << dx^2 /D. However, given that the equation has a term with fourth derivatives in the RHS as well as a non-linear term with sharp gradients. One of these terms should dominate over diffusion to determine the appropriate time step for a finite-difference discretization.*

The purpose of Fig 1 is not to perform a formal stability analysis of the explicit solver but simply to illustrate that customized solvers are more tolerant of larger time steps than standard numerical methods. We realize now that the inclusion of *dt* in Fig 1 is misleading and have substituted its definition with respect to *t_char_* in the revision. In Box 2, we also now expand upon the stiffness of the Cahn-Hilliard equation as follows after Equation 11:

Taking a 3.2 µm x 3.2 µm patch of chromatin with *D* = 10^-3^ µm^2^/second [34], a 2^8^ = 256-element mesh, and *ϵ_4_* ≈ *h* = 3.2 µm/2^8^= 0.0125 µm yields *t_char_* = 0.312 seconds for diffusion across the interface, which limits the time step of the solver, and *t_char_* = 2.84 hours for diffusion across the system, which defines how long the solver must run.

*>4 From a modeling perspective, I also find it curious that the interfacial energy coefficient ϵ_m_ depends on the size of the spatial discretization. From what I understand, this dependency is related to the sharpness of the interface versus mesh resolution. I believe the authors should comment about the sensitivity of the model/models with respect to grid resolution and the continuum limit h → 0 by either performing a sensitivity analysis or by including appropriate references.*

Although *ϵ_m_* may appear to depend on the size of the spatial discretization (*h*), that is just a reference length unit for scaling the number of mesh points that span 90% of an interface. In terms of physical lengths, *ϵ_4_* on a 2^7^ x 2^7^ mesh is identical to *ϵ_8_* on a 2^8^ x 2^8^ mesh, a relationship that we confirm in terms of *R_i_* and *R_eq_* calculations for three values of *m* in Fig 3D,E. In practice, at least three mesh points are needed to span 90% of the interface accurately [4], which is why we only used *m* ≥ 4 here.

*>5 For figure 1: Besides the graphical comparison, the authors should include a quantitative quantification of numerical discrepancies.*

We agree and thank the reviewer for pointing out that our earlier quantification of numerical discrepancies was buried in the supplement. We have revised the main text as follows:

Because NMG and SAV are very different algorithmically (Box 3), it was important to confirm that both packages yield the same numerical solutions. As an initial condition, we used a smoothed random mesh of ±1 chemical states (Fig 1, left; Box 1; and Materials and Methods), which spontaneously undergoes a spinodal decomposition into two phases. After ~1% of characteristic time for the system (*t_char_*; Equation 11), both NMG and SAV converged to a slowly evolving solution with gradually decreasing energy and no overall change in mass (Fig 1A–B and S1 Fig, Panels A–B). The resulting spatial pattern resembles the trajectory of biomolecular condensates triggered to self-assemble on lipid bilayers [44]. We compared results of NMG and SAV to a standard finite-difference implementation of the forward Euler method and successfully captured the first 0.01% of *t_char_* by taking 100-fold smaller time steps (*dt*; Fig 1C). Any larger time steps caused spatial defects in the finite-difference solution along with drastic deviations in energy and mass compared to the true solution (Fig 1D and S1 Fig, Panels A–B). Implicit time steps yielded stable solutions at the expense of overall computation speed (S1 Fig, Panels C–D). NMG and SAV were very similar for alternative boundary conditions and smoothing initializations: between solvers, the root mean squared error was less than 0.02 for unsmoothed spinodal decompositions and less than 0.0125 when initial conditions were smoothed (S1 Fig, Panels E–J). These results verified the self-consistency of our NMG–SAV implementations and reinforced the general need for them.

*>6 Figure 1: The authors compare two distinct implicit/semi-implicit methods to a finite-difference simulation with explicit Euler time evolution. How would these results compare to a simulation using a FD scheme with implicit Euler?*

We implemented a finite-difference scheme with implicit Euler time evolution and added code to the GitHub repository. The method is mass conserving and energy dissipating, even for larger *dt*, and the evolution of the phase field matches the NMG and SAV solvers, but it is twofold slower. We added implicit solver results to S1 Fig, Panels C–D and revised the text as follows:

Because NMG and SAV are very different algorithmically (Box 3), it was important to confirm that both packages yield the same numerical solutions. As an initial condition, we used a smoothed random mesh of ±1 chemical states (Fig 1, left; Box 1; and Materials and Methods), which spontaneously undergoes a spinodal decomposition into two phases. After ~1% of characteristic time for the system (*t_char_*; Equation 11), both NMG and SAV converged to a slowly evolving solution with gradually decreasing energy and no overall change in mass (Fig 1A–B and S1 Fig, Panels A–B). The resulting spatial pattern resembles the trajectory of biomolecular condensates triggered to self-assemble on lipid bilayers [44]. We compared results of NMG and SAV to a standard finite-difference implementation of the forward Euler method and successfully captured the first 0.01% of *t_char_* by taking 100-fold smaller time steps (*dt*; Fig 1C). Any larger time steps caused spatial defects in the finite-difference solution along with drastic deviations in energy and mass compared to the true solution (Fig 1D and S1 Fig, Panels A–B). Implicit time steps yielded stable solutions at the expense of overall computation speed (S1 Fig, Panels C–D). NMG and SAV were very similar for alternative boundary conditions and smoothing initializations: between solvers, the root mean squared error was less than 0.02 for unsmoothed spinodal decompositions and less than 0.0125 when initial conditions were smoothed (S1 Fig, Panels E–J). These results verified the self-consistency of our NMG–SAV implementations and reinforced the general need for them.

>7 Figure 2: The python implementation of the NMG scheme seems to be much slower than the other two implementations, with a discrepancy of three orders of magnitude, while the results for the SAV method seem to be more uniform across different languages. This can be due to slow python loops rather than the algorithm itself. I recommend the authors implement an alternative python version using Cython or specific, pre-compiled numpy functions to accelerate the code in subsequent versions.

We thank the reviewer for this suggestion. We converted the NMG solver to Cython and found that pre-compiling with assigned C data types reduces runtime by 70x–160x for different initial conditions and boundary conditions on a 2^7^x2^7^ mesh. These results are included in Fig 2B of the revision, and the caption is revised as follows:

**Fig 2. Computing performance depends on the programming language for NMG and the boundary condition for SAV. (A)** Snapshots of spinodal decomposition when boundary conditions are periodic (left), whereby flux exiting one edge enters on the opposite edge, or Neumann (right) specifying zero flux at the edges. **(B, C)** Runtime performance for NMG (B) and SAV (C) solvers in Python, MATLAB, and Julia for spinodal decompositions initialized with N = 3 random mixtures of ±1 chemical states (25:75, 50:50, and 75:25) and either periodic or Neumann boundary conditions. For NMG, the Cython implementation is shown relative to Python. For SAV, Neumann boundary conditions require X and Y reflections to re-establish periodic boundaries for the expanded spatial domain (C, inset). Boundary conditions in (C) were tested by multiway ANOVA with boundary condition and programming language as factors.

We also revised the Results text:

In Python, a language that many computational systems biologists consider numerically superior to alternatives (i.e., R), we found that NMG was largely unusable without Cython [45] compilation, requiring 10^5^–10^6^ seconds (by extrapolation) for each simulation (Fig 2B).

*>8 In Figures 2 and 5: The authors state: "All simulations were performed on a Linux x86_64 processor with 100 GB RAM on a 27 x 27 mesh (LX = LY = 1) for 2000 time steps (dt = 5.5e-6) and ϵm=8." Linux is an operating system and x86_64 is a processor architecture. The authors should rephrase this sentence (and similar ones) appropriately and include the actual clock rate (and number of cores used) of the processor.*

Thank you, we updated the information for the following figures:

- Fig 2: All simulations were performed on AMD EPYC 9454 processors (3.81 GHz max, 48 cores per socket, 2 sockets) with up to 300 (Python and MATLAB) or 500 (Julia) GB RAM allocated, using up to 16 cores for computation on a 2^7^×2^7^ mesh for 2000 time steps (dt = 5.5e-6) with ϵm=8.
- Fig 5: All simulations were performed on Intel Xeon Gold 6248 processors (2.50 GHz base, 20 cores per socket, 2 sockets) with 200 GB RAM allocated, on a 2^9^ x 2^9^ mesh (*L_X_* = *L_Y_* = 2) for 26,214 (A–D) or 262,144 (E–H) time steps (*dt* = 1.53e-6).
- S2 Fig: All simulations were performed on AMD EPYC 9454 processors (3.81 GHz max, 48 cores per socket, 2 sockets) with up to 300 (Python and MATLAB) or 1000 (Julia) GB RAM allocated, using up to 16 cores for computation a Linux x86_64 processor with 100 GB RAM on a 2^7^ x 2^7^ mesh (*L_X_* = *L_Y_* = 1) for 2000 time steps (*dt* = 5.5e-6) and *ϵ_m_*_=8_.

*>9 On page 10, the authors state that the source of numerical-stability issues is the fourth-order derivative. This claim does not sound correct, as the fourth order derivative still keeps the diffusive parabolic-like character of the equation. The sharp gradients near the interface combined with the non-linear term would be a much greater source of stiffness, similarly to advection-diffusion-reaction systems.*

We acknowledge the stiffness noted by Reviewer 2 in the sentence immediately following the one about fourth-order derivatives. What we sought to convey in the preceding sentence was that fourth-order derivatives require more mesh refinement to calculate accurately; they also create stiffness challenges for explicit solvers. We have revised this passage as follows:

The energy-minimizing and mass-conserving properties of the Cahn-Hilliard equation set criteria for the accuracy of its numerical solutions. The Cahn-Hilliard equation is intrinsically stiff, because the fast dynamics of interfaces must be considered along with the slower dynamics of phases (Box 2). Additional stiffness challenges arise from the interfacial free energy term embedded within the generalized chemical potential (Equation 7), which results in a fourth-order spatial derivative that is burdensome for standard solvers. Maintaining energy dissipation and mass stability using standard methods requires very-fine spatial meshes, very-small time steps, or global linearizations, any of which adds computational burden.

**REFERENCES**

1.	Tomotika S. On the instability of a cylindrical thread of a viscous liquid surrounded by another viscous fluid. Proceedings of the Royal Society of London A Mathematical and Physical Sciences. 1935;150(870):322–37. doi: 10.1098/rspa.1935.0104.

2.	Trivedi P, Palomba F, Niedzialkowska E, Digman MA, Gratton E, Stukenberg PT. The inner centromere is a biomolecular condensate scaffolded by the chromosomal passenger complex. Nat Cell Biol. 2019;21(9):1127–37. Epub 2019/09/05. doi: 10.1038/s41556-019-0376-4. PubMed PMID: 31481798; PubMed Central PMCID: PMCPMC7341897.

3.	Hedtfeld M, Dammers A, Koerner C, Musacchio A. A validation strategy to assess the role of phase separation as a determinant of macromolecular localization. Mol Cell. 2024;84(9):1783–801 e7. Epub 20240412. doi: 10.1016/j.molcel.2024.03.022. PubMed PMID: 38614097.

4.	Choi JW, Lee HG, Jeong D, Kim J. An unconditionally gradient stable numerical method for solving the Allen-Cahn equation. Physica a-Statistical Mechanics and Its Applications. 2009;388(9):1791–803. doi: 10.1016/j.physa.2009.01.026. PubMed PMID: WOS:000264616900003.
