# Supplementary material for: Cell-specific Cahn-Hilliard models predict condensed fates of the chromosomal passenger complex
Source: PLoS Comput Biol. 2026 Aug 3;22(8):e1014568. doi: 10.1371/journal.pcbi.1014568 (PMC13446744; doi:10.1371/journal.pcbi.1014568)
Supplement: Supplementary file 4 [file pcbi.1014568.s013.docx]

**RESPONSE TO REVIEWERS**

*Reviewer #1: I am satisfied with the authors' responses and am pleased to recommend the manuscript for publication. However, I have one remaining comment regarding Point 1 of the referee report. To clearly demonstrate the t^(1/3) power law in Figure S2, Panels G–H, the authors should present this plot on a log-log scale. This will allow readers to easily discern the specific range over which the power law holds true.*

We have updated these graphs.
