# Supplementary material for: Cell-specific Cahn-Hilliard models predict condensed fates of the chromosomal passenger complex
Source: PLoS Comput Biol. 2026 Aug 3;22(8):e1014568. doi: 10.1371/journal.pcbi.1014568 (PMC13446744; doi:10.1371/journal.pcbi.1014568)
Supplement: Peer review history

# Peer review: Decision Letter 0

**DOI:** 10.1371/journal.pcbi.1014568.r001

**Authors:** Stacey D. Finley, Mohd Suhail Rizvi

12 Mar 2026

PCOMPBIOL-D-25-02313

Cell-specific Cahn-Hilliard models predict condensed fates of the chromosomal passenger complex

PLOS Computational Biology

Dear Dr. Janes,

Thank you for submitting your manuscript to PLOS Computational Biology. Please accept our sincere apologies for the time taken for the review of the manuscript. After careful consideration, we feel that it has merit but does not fully meet PLOS Computational Biology's publication criteria as it currently stands. Therefore, we invite you to submit a revised version of the manuscript that addresses the points raised during the review process.

Please submit your revised manuscript by May 12 2026 11:59PM. If you will need more time than this to complete your revisions, please reply to this message or contact the journal office at ploscompbiol@plos.org. When you're ready to submit your revision, log on to https://www.editorialmanager.com/pcompbiol/ and select the 'Submissions Needing Revision' folder to locate your manuscript file.

Please include the following items when submitting your revised manuscript:

* A letter that responds to each point raised by the editor and reviewer(s). You should upload this letter as a separate file labeled 'Response to Reviewers'. This file does not need to include responses to formatting updates and technical items listed in the 'Journal Requirements' section below.

* A marked-up copy of your manuscript that highlights changes made to the original version. You should upload this as a separate file labeled 'Revised Manuscript with Track Changes'.

* An unmarked version of your revised paper without tracked changes. You should upload this as a separate file labeled 'Manuscript'.

If you would like to make changes to your financial disclosure, competing interests statement, or data availability statement, please make these updates within the submission form at the time of resubmission. Guidelines for resubmitting your figure files are available below the reviewer comments at the end of this letter

We look forward to receiving your revised manuscript.

Kind regards,

Mohd Suhail Rizvi

Academic Editor

PLOS Computational Biology

Stacey Finley

Section Editor

PLOS Computational Biology

**Journal Requirements:**

If the reviewer comments include a recommendation to cite specific previously published works, please review and evaluate these publications to determine whether they are relevant and should be cited. There is no requirement to cite these works unless the editor has indicated otherwise.

1) Please ensure that the CRediT author contributions listed for every co-author are completed accurately and in full.

At this stage, the following Authors/Authors require contributions: Astrid Catalina Alvarez-Yela, Monserrat Gerardo-Ramírez, Sarah Groves, John Lowengrub, Min-Jhe Lu, Todd Stukenberg, and Kevin A. Janes. Please ensure that the full contributions of each author are acknowledged in the "Add/Edit/Remove Authors" section of our submission form.

The list of CRediT author contributions may be found here: https://journals.plos.org/ploscompbiol/s/authorship#loc-author-contributions

2) We have noticed that you have uploaded Supporting Information files, but you have not included a list of legends. Please add a full list of legends for your Supporting Information files after the references list.

**Reviewers' comments:**

Reviewer's Responses to Questions

**Comments to the Authors:**

**Please note here if the review is uploaded as an attachment.**

Reviewer #1: I have attached the report as an attachment.

Reviewer #2: In this work, the authors have built solver libraries for the Cahn-Hilliard equation for Python, MATLAB, and Julia using two different algorithms. The package was then used to explore the problem of condensation of the chromosomal passenger complex on mitotic chromosomes. The simulations results match experimental results. Although the methods here are not necessarily novel, I find the paper to be an interesting read and the open-source algorithms can be useful for other researchers, as phase-field models are becoming increasingly more used in the context biomolecular separation processes. Below, I have some comments, requests for clarifications, and suggestions to the authors that I believe would improve the manuscript.

- Although the title of the paper is related to the specific problem being studied (i.e., CPC condensates), the introduction barely mentions the problem in question or previous related results, some which are outlined later in page 19. The authors should expand the introduction portion of the manuscript to include the problem of CPC condensation, expanding the related bibliography review.

- In page 10, the authors suggest that their CH package is more suitable for biology-focused users, without providing a reason for that claim. I was not able to identify biology-focused features in the solvers in question. The authors should elaborate more on that claim.

- In Figure 1, the authors determine the time step to guarantee stable diffusion dt << dx^2 /D. However, given that the equation has a term with fourth derivatives in the RHS as well as a non-linear term with sharp gradients. One of these terms should dominate over diffusion to determine the appropriate time step for a finite-difference discretization.

- From a modeling perspective, I also find it curious that the interfacial energy coefficient $\epsilon_m$ depends on the size of the spatial discretization. From what I understand, this dependency is related to the sharpness of the interface versus mesh resolution. I believe the authors should comment about the sensitivity of the model/models with respect to grid resolution and the continuum limit $h \to 0$ by either performing a sensitivity analysis or by including appropriate references.

- For figure 1: Besides the graphical comparison, the authors should include a quantitative quantification of numerical discrepancies.

- Figure 1: The authors compare two distinct implicit/semi-implicit methods to a finite-difference simulation with explicit Euler time evolution. How would these results compare to a simulation using a FD scheme with implicit Euler?

- Figure 2: The python implementation of the NMG scheme seems to be much slower than the other two implementations, with a discrepancy of three orders of magnitude, while the results for the SAV method seem to be more uniform across different languages. This can be due to slow python loops rather than the algorithm itself. I recommend the authors implement an alternative python version using Cython or specific, pre-compiled numpy functions to accelerate the code in subsequent versions.

- In Figures 2 and 5: The authors state:

"All simulations were performed on a Linux x86_64 processor with 100 315 GB RAM on a 27 x 27 mesh (LX = LY = 1) for 2000 time steps (dt = 5.5e-6) and ϵm=8."

Linux is an operating system and x86_64 is a processor architecture. The authors should rephrase this sentence (and similar ones) appropriately and include the actual clock rate (and number of cores used) of the processor.

- On page 10, the authors claim state that the source of numerical-stability issues is the fourth-order derivative. This claim does not sound correct, as the fourth order derivative still keeps the diffusive parabolic-like character of the equation. The sharp gradients near the interface combined with the non-linear term would is a much greater source of stiffness, similarly to advection-diffusion-reaction systems.

**********

**Have the authors made all data and (if applicable) computational code underlying the findings in their manuscript fully available?**

The PLOS Data policy requires authors to make all data and code underlying the findings described in their manuscript fully available without restriction, with rare exception (please refer to the Data Availability Statement in the manuscript PDF file). The data and code should be provided as part of the manuscript or its supporting information, or deposited to a public repository. For example, in addition to summary statistics, the data points behind means, medians and variance measures should be available. If there are restrictions on publicly sharing data or code —e.g. participant privacy or use of data from a third party—those must be specified.

Reviewer #1: Yes

Reviewer #2: **No:**  The computational code is available, as described in the Data Availability. However, I did not find a source for the raw experimental data.

**********

PLOS authors have the option to publish the peer review history of their article (what does this mean?). If published, this will include your full peer review and any attached files.

If you choose “no”, your identity will remain anonymous but your review may still be made public.

**Do you want your identity to be public for this peer review?**  For information about this choice, including consent withdrawal, please see our Privacy Policy.

Reviewer #1: **Yes:**  Anupam Gupta

Reviewer #2: No

[NOTE: If reviewer comments were submitted as an attachment file, they will be attached to this email and accessible via the submission site. Please log into your account, locate the manuscript record, and check for the action link "View Attachments". If this link does not appear, there are no attachment files.]

**Figure resubmission:**

While revising your submission, we strongly recommend that you use PLOS’s NAAS tool (https://ngplosjournals.pagemajik.ai/artanalysis) to test your figure files. NAAS can convert your figure files to the TIFF file type and meet basic requirements (such as print size, resolution), or provide you with a report on issues that do not meet our requirements and that NAAS cannot fix.

After uploading your figures to PLOS’s NAAS tool - https://ngplosjournals.pagemajik.ai/artanalysis, NAAS will process the files provided and display the results in the "Uploaded Files" section of the page as the processing is complete. If the uploaded figures meet our requirements (or NAAS is able to fix the files to meet our requirements), the figure will be marked as "fixed" above. If NAAS is unable to fix the files, a red "failed" label will appear above. When NAAS has confirmed that the figure files meet our requirements, please download the file via the download option, and include these NAAS processed figure files when submitting your revised manuscript.

**Reproducibility:**

To enhance the reproducibility of your results, we recommend that authors of applicable studies deposit laboratory protocols in protocols.io, where a protocol can be assigned its own identifier (DOI) such that it can be cited independently in the future. Additionally, PLOS ONE offers an option to publish peer-reviewed clinical study protocols. Read more information on sharing protocols at https://plos.org/protocols?utm_medium=editorial-email&utm_source=authorletters&utm_campaign=protocols

Attachment Submitted filename: Referee_Report.pdf

# Author comment: Author response to Decision Letter 1

**DOI:** 10.1371/journal.pcbi.1014568.r002

11 May 2026

Attachment Submitted filename: GrovesLu_ResponsetoReviewers.docx

# Peer review: Decision Letter 1

**DOI:** 10.1371/journal.pcbi.1014568.r003

**Authors:** Stacey D. Finley, Mohd Suhail Rizvi

5 Jun 2026

PCOMPBIOL-D-25-02313R1

Cell-specific Cahn-Hilliard models predict condensed fates of the chromosomal passenger complex

PLOS Computational Biology

Dear Dr. Janes,

Thank you for submitting your manuscript to PLOS Computational Biology. After careful consideration by the reviewers, we find the revised manuscript to be broadly suitable for publication. However, few  minor issues remain to be addressed before a final decision can be made. Therefore, we invite you to submit a revised version of the manuscript that addresses the points raised during the review process.

Please submit your revised manuscript by Aug 05 2026 11:59PM. If you will need more time than this to complete your revisions, please reply to this message or contact the journal office at ploscompbiol@plos.org. When you're ready to submit your revision, log on to https://www.editorialmanager.com/pcompbiol/ and select the 'Submissions Needing Revision' folder to locate your manuscript file.

Please include the following items when submitting your revised manuscript:

* A letter that responds to each point raised by the editor and reviewer(s). You should upload this letter as a separate file labeled 'Response to Reviewers'. This file does not need to include responses to formatting updates and technical items listed in the 'Journal Requirements' section below.

* A marked-up copy of your manuscript that highlights changes made to the original version. You should upload this as a separate file labeled 'Revised Manuscript with Track Changes'.

* An unmarked version of your revised paper without tracked changes. You should upload this as a separate file labeled 'Manuscript'.

If you would like to make changes to your financial disclosure, competing interests statement, or data availability statement, please make these updates within the submission form at the time of resubmission. Guidelines for resubmitting your figure files are available below the reviewer comments at the end of this letter.

As the corresponding author, your ORCID iD is verified in the submission system and will appear in the published article. PLOS supports the use of ORCID, and we encourage all coauthors to register for an ORCID iD and use it as well. Please encourage your coauthors to verify their ORCID iD within the submission system before final acceptance, as unverified ORCID iDs will not appear in the published article. Only the individual author can complete the verification step; PLOS staff cannot verify ORCID iDs on behalf of authors.

We look forward to receiving your revised manuscript.

Kind regards,

Mohd Suhail Rizvi

Academic Editor

PLOS Computational Biology

Stacey Finley

Section Editor

PLOS Computational Biology

**Reviewers' comments:**

Reviewer's Responses to Questions

**Comments to the Authors:**

**Please note here if the review is uploaded as an attachment.**

Reviewer #1: I am satisfied with the authors' responses and am pleased to recommend the manuscript for publication. However, I have one remaining comment regarding Point 1 of the referee report. To clearly demonstrate the t^(1/3) power law in Figure S2, Panels G–H, the authors should present this plot on a log-log scale. This will allow readers to easily discern the specific range over which the power law holds true.

Reviewer #2: I am satisfied with the authors' responses to my questions as well as the changes made to the manuscript. Therefore, I would be happy to recommend the manuscript for publication.

**********

**Have the authors made all data and (if applicable) computational code underlying the findings in their manuscript fully available?**

The PLOS Data policy requires authors to make all data and code underlying the findings described in their manuscript fully available without restriction, with rare exception (please refer to the Data Availability Statement in the manuscript PDF file). The data and code should be provided as part of the manuscript or its supporting information, or deposited to a public repository. For example, in addition to summary statistics, the data points behind means, medians and variance measures should be available. If there are restrictions on publicly sharing data or code —e.g. participant privacy or use of data from a third party—those must be specified.

Reviewer #1: Yes

Reviewer #2: Yes

**********

PLOS authors have the option to publish the peer review history of their article (what does this mean?). If published, this will include your full peer review and any attached files.

If you choose “no”, your identity will remain anonymous but your review may still be made public.

**Do you want your identity to be public for this peer review?**  For information about this choice, including consent withdrawal, please see our Privacy Policy.

Reviewer #1: **Yes:**  Anupam Gupta

Reviewer #2: No

[NOTE: If reviewer comments were submitted as an attachment file, they will be attached to this email and accessible via the submission site. Please log into your account, locate the manuscript record, and check for the action link "View Attachments". If this link does not appear, there are no attachment files.]

**Figure resubmission:**

While revising your submission, we strongly recommend that you use PLOS’s NAAS tool (https://ngplosjournals.pagemajik.ai/artanalysis) to test your figure files. NAAS can convert your figure files to the TIFF file type and meet basic requirements (such as print size, resolution), or provide you with a report on issues that do not meet our requirements and that NAAS cannot fix.

After uploading your figures to PLOS’s NAAS tool - https://ngplosjournals.pagemajik.ai/artanalysis, NAAS will process the files provided and display the results in the "Uploaded Files" section of the page as the processing is complete. If the uploaded figures meet our requirements (or NAAS is able to fix the files to meet our requirements), the figure will be marked as "fixed" above. If NAAS is unable to fix the files, a red "failed" label will appear above. When NAAS has confirmed that the figure files meet our requirements, please download the file via the download option, and include these NAAS processed figure files when submitting your revised manuscript.

**Reproducibility:**

To enhance the reproducibility of your results, we recommend that authors of applicable studies deposit laboratory protocols in protocols.io, where a protocol can be assigned its own identifier (DOI) such that it can be cited independently in the future. Additionally, PLOS ONE offers an option to publish peer-reviewed clinical study protocols. Read more information on sharing protocols at https://plos.org/protocols?utm_medium=editorial-email&utm_source=authorletters&utm_campaign=protocols

# Author comment: Author response to Decision Letter 2

**DOI:** 10.1371/journal.pcbi.1014568.r004

17 Jun 2026

Attachment Submitted filename: GrovesLu_ResponsetoReviewers2.docx

# Editor report: Decision Letter 2

**DOI:** 10.1371/journal.pcbi.1014568.r005

**Authors:** Stacey D. Finley, Mohd Suhail Rizvi

13 Jul 2026

Dear Dr. Janes,

We are pleased to inform you that your manuscript 'Cell-specific Cahn-Hilliard models predict condensed fates of the chromosomal passenger complex' has been provisionally accepted for publication in PLOS Computational Biology.

Before your manuscript can be formally accepted you will need to complete some formatting changes, which you will receive in a follow up email. A member of our team will be in touch with a set of requests.

Please note that your manuscript will not be scheduled for publication until you have made the required changes, so a swift response is appreciated.

IMPORTANT: The editorial review process is now complete. PLOS will only permit corrections to spelling, formatting or significant scientific errors from this point onwards. Requests for major changes, or any which affect the scientific understanding of your work, will cause delays to the publication date of your manuscript.

Should you, your institution's press office or the journal office choose to press release your paper, you will automatically be opted out of early publication. We ask that you notify us now if you or your institution is planning to press release the article. All press must be co-ordinated with PLOS.

Thank you again for supporting Open Access publishing; we are looking forward to publishing your work in PLOS Computational Biology.

Best regards,

Mohd Suhail Rizvi

Academic Editor

PLOS Computational Biology

Stacey Finley

Section Editor

PLOS Computational Biology

***********************************************************

# Editor report: Acceptance letter

**DOI:** 10.1371/journal.pcbi.1014568.r006

**Authors:** Stacey D. Finley, Mohd Suhail Rizvi

PCOMPBIOL-D-25-02313R2

Cell-specific Cahn-Hilliard models predict condensed fates of the chromosomal passenger complex

Dear Dr Janes,

I am pleased to inform you that your manuscript has been formally accepted for publication in PLOS Computational Biology. Your manuscript is now with our production department and you will be notified of the publication date in due course.

The corresponding author will soon be receiving a typeset proof for review, to ensure errors have not been introduced during production. Please review the PDF proof of your manuscript carefully, as this is the last chance to correct any errors. Please note that major changes, or those which affect the scientific understanding of the work, will likely cause delays to the publication date of your manuscript.

Soon after your final files are uploaded, unless you have opted out, the early version of your manuscript will be published online. The date of the early version will be your article's publication date. The final article will be published to the same URL, and all versions of the paper will be accessible to readers.

For Research, Software, and Methods articles, you will receive an invoice from PLOS for your publication fee after your manuscript has reached the completed accept phase. If you receive an email requesting payment before acceptance or for any other service, this may be a phishing scheme. Learn how to identify phishing emails and protect your accounts at https://explore.plos.org/phishing.

Thank you again for supporting PLOS Computational Biology and open-access publishing. We are looking forward to publishing your work!

With kind regards,

Sharmila Kamatchi

PLOS Computational Biology | Carlyle House, Carlyle Road, Cambridge CB4 3DN | United Kingdom ploscompbiol@plos.org | Phone +44 (0) 1223-442824 | ploscompbiol.org | @PLOSCompBiol
